# Chitosan-dextran sulfate nanocapsules for enhanced tigecycline efficacy against non-typhoidal *Salmonella enterica*

**DOI:** 10.1038/s41598-026-35229-7

**Published:** 2026-02-04

**Authors:** Mona R. Omar, Ahmed A. Saeed, Seham M. Malhat, Ahmed A. El-Sayed, Abdelmoneim A. Ali, Christina R. B. Youssef, Yasmine H. Tartor

**Affiliations:** 1 Animal Health Research Institute, Zagazig, Egypt; 2 Department of Pharmacology, Faculty of Veterinary Medicine, Zagazig University, Zagazig, 44511 Egypt; 3 Photochemistry Department, Industrial Chemical Research Institute, National Research Center, 12622 Dokki, Giza Egypt; 4 Department of Pathology, Faculty of Veterinary Medicine, Zagazig University, Zagazig, 44511 Egypt; 5 Department of Microbiology and Immunology, Faculty of Pharmacy, Zagazig University, Zagazig, 44519 Egypt; 6 Department of Microbiology, Faculty of Veterinary Medicine, Zagazig University, Zagazig, 44511 Egypt

**Keywords:** *Salmonella enterica*, Non-typhoidal *Salmonella*, Chitosan, Dextran sulfate nanocapsule, Tigecycline, Anti-efflux, Resistance genes, Drug delivery, Microbiology, Nanoscience and technology

## Abstract

*Salmonella* is a significant foodborne pathogen found worldwide. Resistance to tigecycline (TGC) has been increasingly reported. The emergence of multidrug-resistant (MDR) non-typhoidal *Salmonella* (NTS) with high efflux pump activity necessitates the development of efficient drug delivery systems. This study investigates the potential of tigecycline-loaded chitosan-dextran sulfate (CD-TGC) nanocapsules to combat NTS in vitro and in vivo mouse peritonitis model. Antimicrobial susceptibility and efflux pump activity of NTS isolates were tested. *S. enterica* serotype Bredeney isolates that showed high efflux index and high multiple antibiotic resistance index were subjected to whole genome sequencing (WGS), revealing numerous resistance genes, including APH(3’’), *Mar*A, *Ma*rB, *Mar*R, *Alr*, *Ddl*, *dxr*, *Bcr*C, AcrAB-TolC, AcrAD-TolC, *gid*B, *Gdp*D, *Pgs*A, H-NS, and *Oxy*R. Additionally, the presence of resistance-nodulation-cell division (RND) efflux pumps, major facilitator superfamily (MFS), and ATP-binding cassette (ABC) efflux pumps were confirmed. CD-TGC demonstrated significantly lower minimum inhibitory concentrations (MIC 0.5–1 µg/mL) against NTS strains compared to tigecycline (TGC) alone (MIC 32–128 µg /mL). CD-TGC decreased MIC 7-fold, from 128 to 1 µg/mL, in one *S.* Typhimurium strain. In the other 11 strains, CD-TGC reduced the TGC MIC 6-fold, from 64 to 1 µg/mL (*n* = 6) and 32 to 0.5 µg/mL (*n* = 5). Time-kill assays confirmed enhanced bactericidal activity. Furthermore, CD-TGC downregulated the expression of *ramA* and *acrB* efflux pump genes. In a mouse model of *S.* Typhimurium infection, CD-TGC treatment effectively reduced bacterial burden in the liver and intestine, minimized liver and kidney function alterations, and decreased mortality rates compared to tigecycline and unloaded CD nanocapsules. The survival rates of mice were 100% in the CD-TGC treatment group and 40% in the TGC treatment group. Histopathological analysis confirmed reduced tissue damage in the CD-TGC-treated group. The findings indicate that CD-TGC nanocapsules offer a promising drug delivery strategy for treating intracellular *Salmonella* infections, overcoming resistance mechanisms and improving treatment outcomes where tigecycline alone is less effective.

**Supplementary Information:**

The online version contains supplementary material available at 10.1038/s41598-026-35229-7.

## Introduction

*Salmonella* is an important zoonotic pathogen causing substantial economic losses. Non-typhoidal *Salmonella* (NTS) serovars are the primary cause of both intestinal and extra-intestinal infections^1^. NTS is a major bacterial cause of diarrhea worldwide, resulting in approximately 150 million illnesses and 60,000 deaths annually^2^. In low-and middle-income countries, bloodstream infections caused by *Salmonella enterica* serovars are most common, and serious complications such as meningitis and gastrointestinal perforation can arise from *Salmonella* infections when antibiotic treatment is ineffective^3^. The increasing prevalence of antimicrobial-resistant *Salmonella*, particularly in chicken meat contaminated with NTS, is a major public health concern worldwide^4^.

Due to the widespread use of antimicrobials in humans and livestock production, *Salmonella* has developed increasing resistance to antimicrobials such as β-lactams, aminoglycosides, sulfonamides, tetracyclines, and the quinolones^5^. A broad-spectrum antibiotic for combating *Salmonella* infections is tigecycline (TGC), which has gained attention as a last-resort treatment^6^. A major concern is the increasing tetracycline resistance in *Salmonella*, which has been linked to the unreasonable overuse of TGC and has attracted widespread attention^7^. Bacterial efflux pump systems are crucial in the development of TGC resistance. Notably, AcrAB-TolC is a prominent multidrug-resistant efflux pump belonging to the resistance-nodulation-division (RND) family^7–10^. The challenge of treating some intestinal infections stems from poor drug penetration into host cells and inadequate delivery to infection sites. Improved drug delivery, selectivity, and macrophage-targeted intracellular transport are urgently needed^11^.

Prolonging antibiotic contact with microbial surfaces enhances the treatment of infectious diseases. Nanotechnology alters the characteristics of nanoparticles utilized as antimicrobial agents, drug delivery methods, cellular labeling, biomarkers, diagnostic tools, bioimaging techniques, and nanotherapeutics for the treatment of many diseases^12^. Furthermore, nanocarriers have been utilized in the management of bacterial infections^13^. The delivery carriers may be micro- or nano-sized and comprise peptides, polymers, liposomes, nanoparticles, and numerous other inorganic nanomaterials^14^. Adsorption, encapsulation, and chemical conjugation can all be used to insert the medications into nanocarriers, which will enable them to efficiently and very specifically reach the intended site^15^. Nanovehicle delivery of antibiotics protects the drugs from bacterial resistance mechanisms and, if the nanovehicle itself has antibacterial properties, allows for lower antibiotic dosages and concentrations^16^. Nanovehicles can also improve drug pharmacokinetics, extend drug retention, enhance biocompatibility, and reduce harm to the host^17^. Ongoing research into new antimicrobials has yielded antimicrobial biomaterials based on polymers and their composites^18^. Polymeric nanoparticles can associate with negatively charged bacterial membranes, enhancing their permeability^19^. Chitosan (CH) is a biodegradable cationic polymer known as (1, 4)-2-amino-2-deoxy-D-glucan. This linear polyamino saccharide, derived from the N-deacetylation of chitin, is biocompatible and mucoadhesive, suggesting its potential as a drug carrier^20^. Furthermore, it exhibits enhanced antibacterial and antibiofilm properties^21^. However, CH alone does not possess targeted macrophage specificity for treating pathogenic intracellular *S*. Typhi. Targeted delivery to intracellular infection sites, achieved by conjugating CH with a carbohydrate polymer, is necessary to enhance its efficacy^20^. The usefulness of CH is restricted as it is ineffective at an acidic pH^22^.

To address this problem, dextran sulfate (DS), a biocompatible polyanionic polymer and branched-chain polysaccharide characterized by 1–6 and 1–4 glycosidic linkages, has been utilized for its significant function in enzyme inhibition and as a drug conjugate for drug delivery^23^.

No previous comprehensive study has examined the anti-efflux activity of tigecycline-loaded chitosan-dextran sulfate (CD-TGC) nanocapsules against *S. enterica* strains and the genetic features of *S. enterica* serotype Bredeney strains with high efflux pump activity circulating in Egypt. This study was designed to: (i) characterize the genetic features of NTS exhibiting high efflux pump activity and harboring resistance genes, (ii) investigate the anti-efflux pump activity of CD-TGC against NTS, and (iii) in vivo activity of CD-TGC against *S*. Typhimurium infection in mice model.

## Materials and methods

### *Salmonella enterica* strains

This study included twelve *Salmonella enterica* strains from chicken and duck meat samples that were purchased from poultry retail outlets at Zagazig, Egypt (Table 1). Strains were identified through biochemical characters, including oxidase, methyl red, Voges–Proskauer, and citrate utilization tests, along with their unique reactions on triple sugar iron agar and lysine decarboxylase media (Oxoid, United Kingdom). *Salmonella* strains were serotyped by commercially available antisera (Denka Seiken Co., Ltd., United Kingdom) following the antigenic profile defined by Kauffmann^24^. Polymerase chain reaction (PCR) of the *invA* gene was performed to confirm *Salmonella* identification^25^.

**Table 1:** Antimicrobial resistance profile and efflux pump activity of *Salmonella enterica* strains included in the study.

Serovar	Source	Antimicrobial resistance pattern	MAR index	MIC (µg/mL)				After TGC treatment		After CD-TGC treatment	
				TGC	CIP	CT	CD-TGC nanocapsule	Efflux pump activity	MC_EtBr_ (µg/mL) index	Efflux pump activity	MC_EtBr_ (µg/mL) index
*S*. Bredeney (code no 33)	Duck	AMC, SAM, CZ, CXM, FEB, FOX, CN, TOB, CIP, TE, TGC, FOS, C, SXT, CT, NA, IPM, ETP, AM.	0.79	128	2	4	1	2.5	9	1.5	5
*S*. Bredeney (code no 5)	Chicken	AM, AMC, SAM, CZ, CXM, CRO, FEB, FOX, CN, AK, CIP, TE, TGC, FOS, C, SXT, ATM, CT	0.75	64	2	32	1	2.5	9	1	3
*S.*Typhimurium	Chicken	AM, AMC, SAM, CZ, CX, CRO, FEB, FOX, TOB, NA, CIP, TE, TGC, FOS, C, SXT, CT	0.71	64	16	16	1	2	7	1.5	5
*S.*Typhimurium	Chicken	AM, AMC, SAM, CZ, CXM, CRO, FEB, FOX, TOB NA, CIP, TE, TGC, FOS, C, SXT, CT	0.71	64	8	16	1	2	7	1	3
*S.* Magheroftt	Chicken	AM, AMC, SAM, CZ, CXM, CRO, FEB, FOX, TOB, CIP, TE, TGC, FOS, C, SXT, CT	0.67	32	16	8	0.5	1.5	5	0.5	1
*S.* Typhimurium	Chicken	AM, AMC, SAM, CZ, CXM, CRO, FEB, FOX, TOB, NA, CIP, TE, TGC, FOS, C, SXT, CT	0.71	32	2	32	0.5	1.5	5	0.5	1
*S.* Takoradi	Chicken	AM, AMC, SAM, CZ, CXM, CRO, FEB, FOX, CN, TOB, CIP, TE, TGC, FOS, C, SXT, CT	0.71	32	8	8	0.5	1.5	5	0.5	1
*S.*Typhimurium	Chicken	AM, AMC, CZ, CXM, CRO, FEB, FOX, CN, AK, CIP, TE, FOS, C, SXT, ATM, CT, TGC	0.71	64	4	8	1	2.5	9	1	3
*S.* Bredeney	Chicken	AM, AMC, SAM, CZ, CXM, CRO, FEB, FOX, TOB, NA, CIP, TE, TGC, FOS, C, SXT, CT, IPM, ETP.	0.71	64	32	32	1	2	7	1.5	5
*S.*Typhimurium	Chicken	AM, AMC, SAM, CZ, CXM, CRO, FEB, FOX, TOB, NA, CIP, TE, TIG, FOS, C, SXT, CT	0.71	64	8	16	1	2	7	1	3
*S.* Enteritidis	Chicken	AM, AMC, CZ, CXM, CRO, FOX, ETP, IPM, CN, TOB, CT CIP, TE, C, SXT, TGC	0.67	32	16	8	0.5	1.5	5	0.5	1
*S.*Typhimurium	Chicken	AM, AMC, SAM, CZ, CXM, CRO, FEB, FOX, TOB, NA, CIP, TE, TGC, FOS, C, SXT, CT	0.71	32	8	16	0.5	1.5	5	0.5	1

MIC, minimum inhibitory concentration; AM, ampicillin; AMC, amoxicillin-clavulanic acid; SAM, ampicillin-sulbactam; CZ, cefazolin; CXM, cefuroxime; CRO, ceftriaxone; FEB, cefepime; FOX, cefoxitin; IPM, imipenem; ETP, ertapenem; CN, gentamicin; TOB, tobramycin; AK, amikacin; CIP, ciprofloxacin; TE, tetracycline; TIG, tigecycline; FOS, fosfomycin; C, chloramphenicol; SXT, sulfamethoxazole-trimethoprim; ATM, aztreonam; CT, colistin; MEM, meropenem; DOR, doripenem; NA, nalidixic acid; MAR, multiple antibiotic resistance index; CD-TGC, tigecycline-loaded chitosan-dextran sulfate nanocapsule; MC_EtBr_, minimal concentration of ethidium bromide that induces fluorescence in the test strain.

### Antimicrobial susceptibility testing of *Salmonella* strains

The antimicrobial susceptibilities of all *Salmonella enterica* strains were assessed against 24 antibiotics (Oxoid, Hampshire, England, United Kingdom) using the disk diffusion method^26^. The tested antimicrobial agents were amoxicillin-clavulanic acid (AMC, 20 µg/10 µg), ampicillin-sulbactam (SAM, 20/10 µg), cefazolin (CZ, 30 µg), ceftriaxone (CRO, 30 µg), cefuroxime (CXM, 30 µg), cefepime (FEB, 30 µg), cefoxitin (FOX, 30 µg), imipenem (IPM, 10 µg), ertapenem (ETP, 10 µg), meropenem (MEM, 10 µg), doripenem (DOR, 10 µg), ciprofloxacin (CIP, 5 µg), trimethoprim-sulfamethoxazole (SXT, 1.25 µg/23.75 µg), tetracycline (TE, 30 µg), ampicillin (AM, 10 µg), aztreonam (ATM, 30 µg), gentamicin (GM, 10 µg), tobramycin (10 µg), amikacin (AK, 30 µg), nalidixic acid (NA, 30 µg), tigecycline (TIG, 15 µg), fosfomycin (FOS, 50 µg), colistin (CT, 10 µg), and chloramphenicol (C, 30 µg). The inhibition zone diameters were interpreted in accordance with the guidelines of the Clinical and Laboratory Standards Institute (CLSI) and the European Committee on Antimicrobial Susceptibility Testing (EUCAST)^27,28^. The minimal inhibitory concentrations (MICs) of TGC, CT, and CIP were determined by broth microdilution method using the CLSI and EUCAST interpretive criteria. The MIC breakpoints used were: TGC > 2 µg/mL, CT > 2 µg/mL, and CIP ≥ 1 µg/mL.^27, 28^ The multiple antibiotic resistance (MAR) index was calculated as previously described^29^.

### Testing efflux pump activity

The efflux pump activity of *Salmonella* strains was assessed using the ethidium bromide (EtBr) cartwheel method^30^. On the day of the experiment, trypticase soy agar (TSA, Oxoid, United Kingdom) plates containing ethidium bromide (EtBr, Sigma-Aldrich, Germany) at concentrations 0.0, 0.5, 1.0, 1.5, 2.0, and 2.5 mg/L were prepared. Each *Salmonella* strain (1.5 × 10^8^ colony-forming units (CFU)/mL) was inoculated on an EtBr plate in a cartwheel arrangement. The plates were enveloped in aluminum foil and incubated at 37 °C for an overnight period. The lowest concentration of EtBr that elicited fluorescence in bacterial colonies under the Accuris TM E3000 UV Transilluminator (Accuris Instruments, United States) was documented. A sensitive *Salmonella* strain (*S*. Virchow) served as a comparative control for fluorescence analysis. The ability of each *Salmonella* strain to extrude EtBr substrate was assessed in comparison to the control isolate using the following equation:

$${\text{Efflux activity index }}=~\frac{{~M{C_{EtBr(MDR)}} - M{C_{EtBr}}\left( {REF} \right)}}{{M{C_{EtBr}}~\left( {REF} \right)}}$$

where ${\mathrm{M}}{{\mathrm{C}}_{{\mathrm{EtBr}}}}$ is the minimal concentration of EtBr that induces fluorescence in the test strain. Simultaneously${\mathrm{M}}{{\mathrm{C}}_{{\mathrm{EtBr}}}}$ (REF) denotes the minimal concentration of EtBr required to elicit fluorescence in the reference strain.

### Whole genome sequencing

The strains were cultured overnight at 37 °C on tryptone soya broth (TSB, Oxoid, United Kingdom) and bacterial DNA was extracted using GenElute Bacterial Genomic DNA kit according to the manufacturer’s instructions (Sigma-Aldrich). The sequence was performed using Oxford Nanopore technology using the rapid barcoding kit 96 SQK-RBK 110.96. Complete genome analysis was performed using the comprehensive genome analysis service in PATRIC web resources^31^. Multi Locus Sequence Typing (MLST) was performed using MLST 2.0.9 detection service^32^. PlasmidFinder 2.1 was used to identify plasmids^33^. Resistance genes were identified by Resistance Gene Identifier (RGI) on the Comprehensive Antibiotic Resistance Database (CARD) (https://card.mcmaster.ca/home). The sequences were submitted to the NCBI Sequence Read Archive (SRA) (https://submit.ncbi.nlm.nih.gov/subs/sra/) with BioProject accession number PRJNA1189127.

### Synthesis of cross-linked chitosan–dextran sulfate nano-dispersion and nanoparticles encapsulated Tigecycline

Tigecycline was obtained from Tygacil^®^, Pfizer Inc., and chitosan (low molecular weight) was obtained from Sigma Aldrich Chemicals, Germany. Dextran sulfate, acetic acid, and all other substances were of analytical reagent quality and procured from Sigma Aldrich Fine Chemicals. Various formulation batches were conducted to assess the impact of formulation variables on the dependent variables. The optimization of the formulation employed a central composite rotatable design to examine the effects of independent factors, specifically chitosan and DS concentrations, on the dependent variables, mean particle size (MPS) and Zeta potential. The four distinct batches of lyophilized nanoparticles were formulated with 50 mg of chitosan, 12.5 mg of DS, a MPS of 129.4 nm, a polydispersity index (PDI) of 0.125, and a zeta potential of 51.3 mV.

**Fig. 1:**
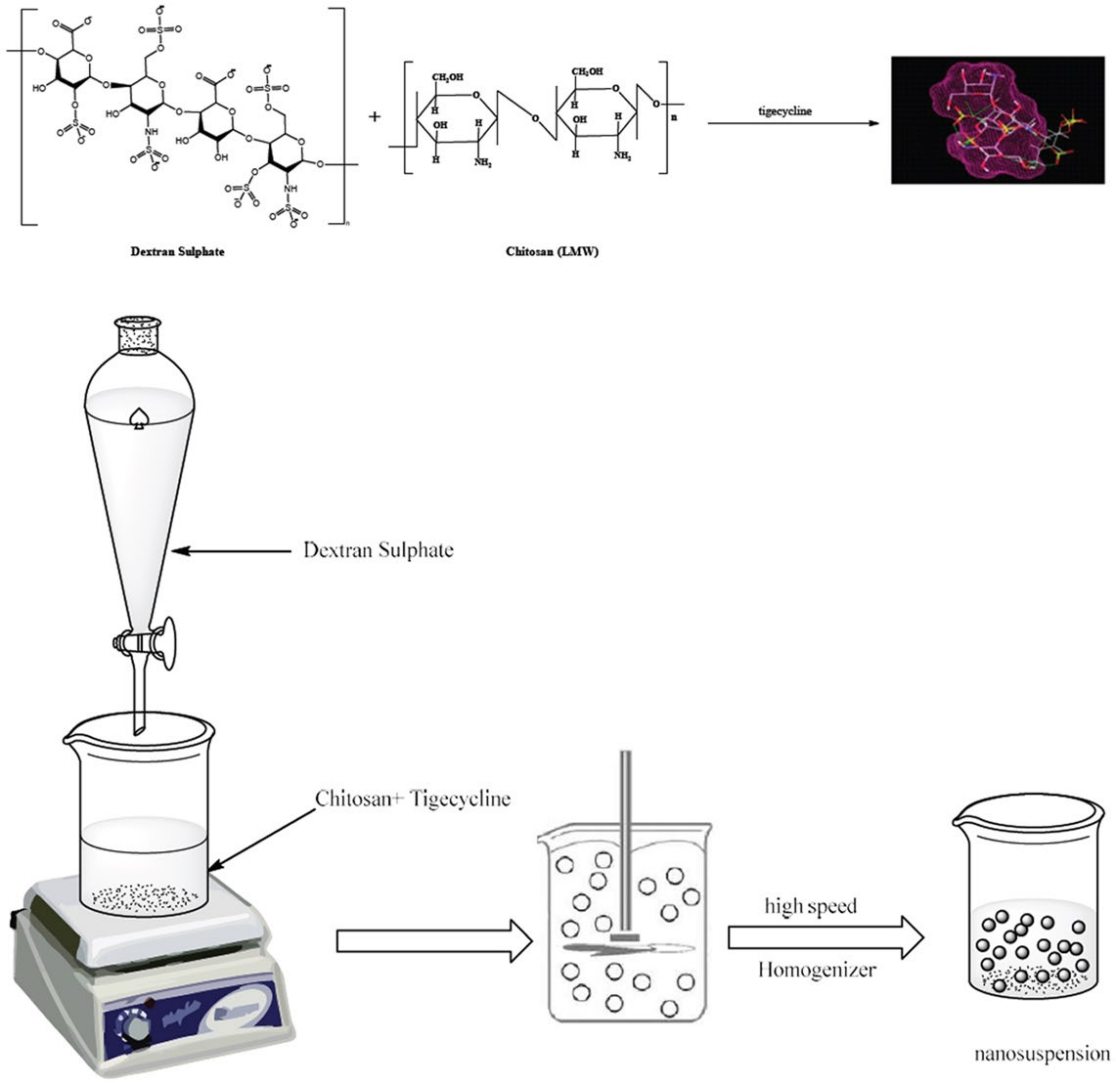
Preparation of tigecycline -loaded nanoparticles using dextran sulphate and low molecular weight (LMW) chitosan.

Low molecular weight chitosan (0.5 g) was dissolved in 80 mL of 2% aqueous acetic acid solution while stirring until complete dissolution occurred (Fig. 1). Tigecycline (50 mg) was diluted in 10 mL of distilled water, incorporated into the chitosan solution with continuous stirring for approximately one hour, and subjected to sonication for an additional hour. Dextran sulfate (0.125 g) was dissolved in 10 mL of distilled water (ratio 8:2) and incrementally added to the chitosan mixture with continuous agitation at ambient temperature. The solution transformed into a milky suspension, signifying the synthesis of chitosan nanoparticles^34^.

### Characterization of chitosan/dextran sulfate nanoparticles encapsulated Tigecycline

#### Transmission electron microscopy (TEM)

The dimensions and morphology of CD NPs were effectively determined using High-Resolution Transmission Electron Microscopy (HRTEM) (JEOL-JEM 2100, Japan). For transmission electron microscopy (TEM) observations, a droplet of colloidal solution was deposited onto a 400-mesh carbon-coated copper grid, and the solvent was allowed to evaporate in ambient air at room temperature.

#### Distribution of particle size and zeta potential

The average diameter, size distribution, and zeta potential of the samples were evaluated using a particle size analyzer (Nano-ZS, Malvern Instruments Ltd., UK). The sample was subjected to sonication for 10–20 min before the measurement of size distribution and zeta potential.

#### X-ray powder diffraction (XRD)

Powder diffraction was conducted using Cu (λ = 1.54 Å) with a Ni filter at room temperature (voltage = 40 kV) employing a Brucker Axs, 08 Advance X-ray powder diffractometer. Analyses were conducted on samples of pure fenofibrate (FBT), poloxamer 188, a physical combination of FBT and poloxamer 188, and lyophilized nanocrystals. The equipment was utilized at a scanning rate of 1°/min for a 2θ of 70°.

#### Stability of tigecycline-loaded chitosan-dextran sulfate nanocapsules

The kinetic release of TGC from nano-encapsulation was examined in 0.1 M phosphate-buffered saline (PBS) solution at a pH 7.4 which mimics the pH of blood. A dialysis bag with a molecular weight cut of (12–14 KDa) was used to absorb 60 mg of the nano-encapsulation medication, which had an initially high concentration, in a 2 mL buffer solution. The bag was subsequently immersed in a 30 mL PBS reservoir with gentle agitation (at 100 rpm). To facilitate TGC release from the nano-encapsulation, 1 mL of the phosphate buffer reservoir was removed at predetermined intervals and replaced with a new 1 mL buffer. A UV spectrophotometer (PerkinElmer, Inc., Waltham, Massachusetts, USA) operating at 245 nm was used to ascertain the dosage of the discharged TGC ^35.^

#### Cumulative amount of TGC released (mg)

Since each sample is 1 mL, the amount of TGC in each withdrawn sample = Conc. × 1 mL. However, since we are sampling and replacing the buffer, we must account for the cumulative release (i.e., keep track of the drug lost from the reservoir and replaced by zero-concentration buffer). This recursive formula was used:

$${\mathrm{Q}}_{{\mathrm{t}}} = {\text{ C}}_{{\mathrm{t}}} {\text{X V }} + \sum\limits_{{i = 1}}^{{t - 1}} {{\mathrm{C}}_{{\mathrm{i}}} {\text{X V}}_{{\mathrm{s}}} }$$

Q_t_ refers to cumulative drug amount released at time t, C_t_: concentration at time t, C_i_: concentration at previous time points, V: total volume of dissolution medium, and V_s_ represents volume of sample withdrawn at each time point.

#### Drug loading onto Chitosan dextran sulphate

Tigecycline nano-encapsulated with the lowest particle size and a reasonable negative zeta potential was selected to be used in drug loading. Tigecycline was dissolved in distilled water and added to the CH low molecular weight in 1% acetic acid, and then DS was added drop by drop, stirred for an hour, then centrifuged at 6000 rpm for 30 min. The solution removed from the centrifuge was analyzed by UV–Vis spectroscopy (PerkinElmer, Inc., Waltham, Massachusetts, USA) to determine the concentration of unloaded drug. The drug loading content (DL) and encapsulation efficiency (EE) were evaluated as follows:

$${\mathrm{DL}}\% {\text{ }}={\text{ }}\left( {{\text{Weight of the drug in encapsulation }}/{\text{Weight of the chitosan dextran sulphate}}} \right) \times {\mathrm{1}}00$$

$${\mathrm{EE}}\% ={\text{ }}\left( {{\text{Weight of the drug in encapsulation }}/{\text{Weight of the feeding drugs}}} \right) \times {\mathrm{1}}00$$

#### Fitting drug release kinetics models

We’ll try to fit:

1. Zero-order: *Q*_*t*_ *= Q*_*0*_ *+ k*_*0*_*t*.
2. First-order: *log Q*_*t*_ *= log Q*_*0*_ *− k*_*1*_*t/2.303​*.
3. Higuchi: Q_t_ =k_H_ √t​.
4. Korsmeyer-Peppas: *Q*_*t*_*/Q*_*∞*_*=kt*^*n*^.

### Safety of tigecycline-loaded chitosan-dextran sulfate nanocapsules on BJ normal human fibroplasts

BJ normal human foreskin primary fibroblast cell line (ATCC CRL-2522) was used for studying safety of CD-TGC nanocapsules. Cell line viability was assessed by the mitochondrial-dependent reduction of yellow MTT (3-(4,5-dimethylthiazol-2-yl)-2,5-diphenyl tetrazolium bromide) to purple formazan^36^. All procedures were done in a sterile area using a Laminar flow cabinet biosafety class II level (Baker, SG403INT, Sanford, ME, USA). BJ normal human foreskin fibroblasts were suspended in Dulbecco′s Modified Eagle′s Medium/Nutrient Mixture F-12 Ham medium containing 1% antibiotic mixture (10,000 U/mL potassium penicillin, 10,000 µg/mL streptomycin sulfate and 25 µg/mL amphotericin B) and 1% L-glutamine (Sigma-Aldrich, Germany) at 37 °C under 5% CO_2_. Cells were batch cultured for 10 days, then seeded at a concentration of 10 × 10^3^ cells/well in fresh complete growth medium in 96-well microtiter plastic plates at 37 °C for 24 h under 5% CO_2_ using a water jacketed Carbon dioxide incubator (Sheldon, TC2323, Cornelius, OR, USA). Media was aspirated, fresh medium (without serum) was added, and cells were incubated either alone (negative control) or with different concentrations of sample to give a final concentration of 100, 50, 25, 12.5, 6.25, 3.125, 0.78, and 1.56 µg/mL). After 48 h of incubation, the medium was aspirated, 40 µL MTT salt (2.5 µg/mL) was added to each well, and incubated for a further four hours at 37 °C under 5% CO_2_. To stop the reaction and dissolve the formed crystals, 200 µL of 10% Sodium dodecyl sulphate (SDS) in deionized water was added to each well and incubated overnight at 37°C. DOX was used as a positive control at 100 µg/mL gives 100% lethality under the same conditions^37^.

The absorbance was then measured using a microplate multi-well reader (Bio-Rad Laboratories Inc., model 3350, Hercules, California, USA) at 595 nm and a reference wavelength of 620 nm. DMSO is the vehicle used for dissolution the nanocapsule, and its final concentration on the cells was less than 0.2%. The percentage of change in viability was calculated according to the formula: [(Reading of tested sample​/Reading of negative control​) − 1]×100.

A probit analysis was carried out using IBM SPSS Statistics to determine the lethal concentration of the sample at which 50% (IC50) and 90% (IC90) of cells die within 48 h. Statistical significance was tested between samples and negative control (cells with vehicle) using an independent t-test.

### Time-kill curves

Suspensions of *S*. Typhimurium were diluted to approximately 8 × 10^4^ and 8 × 10^6^ CFU/mL in 25 mL of Mueller-Hinton broth (Oxoid, United Kingdom) in separate 125-mL glass conical flasks. Susceptible breakpoint concentrations of TGC (2 µg/mL) were prepared and placed in flasks^38^. Each flask was incubated at 37 °C. The bacterial count was assessed at 0, 4, 8, 24, and 48 h by counting the colonies in 100-µL aliquots of 10-fold serially diluted specimens plated on XLD agar medium (Oxoid Ltd., Hampshire, UK).

### Expression of efflux pump genes

SYBR Green real-time PCR was employed to assess the relative expression levels of the *ram*A and *acr*B genes using the oligonucleotide primers: ramA-F (5′-CACGATTGTCGAGTGGATTG − 3′) and ramA-R (5′-AAAATGCGCGTAAAGGTTTG − 3′)^39^ and acrB-F (5′-GGCATTGGGTATGACTGGAC-3′) and acrB-R (5`-GCATTACGGAGAACGGGATAG-3`),^40^ with the 16S rRNA gene: 16S rRNA-F(5′-CAGAAGAAGCACCGGCTAACTC-3′) and 16S rRNA-R (5`-GCGCTTTACGCCCAGTAATT-3`) serving as the housekeeping gene^41^. Total RNA was extracted from *Salmonella* strains using the QIAamp RNeasy Mini kit (Qiagen, Germany) in accordance with the manufacturer’s guidelines. Relative quantification was performed using the Quantitect SYBR Green PCR kit (Qiagen, Germany) in the MX3005P real-time PCR thermal cycler (Agilent, La Jolla, CA, United States), adhering to the manufacturer’s guidelines. The Stratagene MX3005P program was utilized to ascertain amplification curves and CT values. To evaluate the differential gene expression of RNA among various samples, the CT values of each sample were compared with those of the control group utilizing the “^ΔΔ^CT” method as delineated by Yuan et al.^42^. The possibility of false positive results was eliminated by comparing dissociation curves across many samples.

### In vivo evaluation using mice peritonitis model

Fifty mice were randomly assigned to five groups of ten mice each. The mice were obtained from the animal facility of the Faculty of Veterinary Medicine at Zagazig University. The animals underwent a 10-day acclimation period and were fed a high-quality commercial balanced diet and water ad libitum.

Mice were fasted for 12 h before bacterial inoculation. Four groups of mice were infected with *S.* Typhimurium intraperitoneally at a dose of 1.3 × $\:{10}^{6}$ CFU/mL, and 0.1 mL of the diluted culture was injected into the peritoneal cavity (i.p.), and the fifth group (control negative) received PBS. Six hours post-infection, three groups received subcutaneous injections every 12 h for 4 days of either TGC (6.25 mg/kg),^38^ CD-TGC (25 mg/kg), or unloaded CD NPs (6.25 mg/20 gm). The positive control group was infected with *S. typhimurium* but received no treatment. The negative control group received PBS only. The dose of CD-TGC was determined based on the lethal dose 50 (LD50)^43^. Animals were examined twice daily for signs of illness or death for 15 days. Five mice from each group were sacrificed after 4 days. At the end of the experiment, euthanasia was performed with pentobarbital sodium i.p. (120 mg/kg body weight), followed by cervical dislocation for confirmation.

This study protocol, including all animal procedures, was performed following the ARRIVE guidelines and regulations (https://arriveguidelines.org) and was approved by the Zagazig University Institutional Animal Care and Use Committee (ZU-IACUC) under approval number ZU-IACUC/2/F/12/2019.

#### Measurement of biochemical and hematological parameters

To assess clinical chemistry parameters, blood samples were collected from mice, allowed to clot, and then centrifuged at 3500 rpm for 5 min to obtain serum. The serum total protein (TP), albumin (Alb), aspartate aminotransferase (AST), alanine transaminase (ALT), lactate dehydrogenase (LDH), total bilirubin, urea, and creatinine levels were measured in various animal groups using Spinreact kits (Esteve De Bas, Girona, Spain) according to manufacturer instructions.

Hematological parameters, including hemoglobin (Hb) and white blood cell (WBC) counts, were assessed using a cell blood counter (Celltac Alpha MEK6550, Nihon Kohden Company, Japan). A Giemsa-stained blood smear was used to differentiate WBCs into neutrophils and lymphocytes.

#### Assessment of bacterial load

The liver and intestine were collected, weighed, and homogenized in sterile PBS. The homogenate was serially diluted and plated on XLD agar. CFUs per gram of organ were counted after a 24-hour incubation at 37 °C.

#### Histopathological analysis

The specimens obtained from the liver and intestine of various groups were preserved in 10% neutral buffered formalin, dehydrated through increasing concentrations of alcohol, cleaned with xylene, and embedded in molten paraffin wax. Five-micrometer paraffin slices were prepared using a microtome (Thermo Scientific, Massachusetts, USA) and subsequently stained with hematoxylin and eosin (H&E)^44^.

Histopathological assessments were performed by investigators blinded to group assignments. A semiquantitative scoring system was employed, following the ordinal method^45^. Hepatic and intestinal injury was scored by examining three fields per rat (six rats per group) in photographed H&E-stained sections at 400× magnification. Hepatic damage was evaluated through hepatocyte degradation and necrosis accompanied by mononuclear cell infiltration. Intestinal injury was evaluated based on necrosis of villi, cystic gland formation, and submucosal edema. The semiquantitative analysis was scored on four grades: 0, indicating no pathological changes; 1, representing mild alterations; 2, denoting moderate changes; and 3, signifying severe pathological abnormalities^46^.

### Data analysis

Shapiro–Wilk test for normality and Levene’s test for homogeneity of variance were conducted on all numerical data. Data analysis was performed with SPSS version 29.0 (IBM Corp., Armonk, NY, USA). Blood parameters were analysed by Analysis of Variance (ANOVA), with Tukey’s range test applied to determine differences among group means^47^. All experiments were performed in triplicate, with three independent biological replicates, and results are expressed as mean ± standard error (SE). Multiple ANOVAs were performed to evaluate the impacts of various groups, incorporating two between-subjects factors and one within-subjects factor. Kaplan–Meier survival curves with log-rank test was employed to compare the survival rates among the various animal groups. The results were considered statistically significant when the *p* value was less than 0.05. The histopathological findings from the investigated groups were analyzed using one-way ANOVA followed by Duncan’s post hoc test.

## Results

### Antimicrobial susceptibility of *Salmonella enterica* strains to different antimicrobials

*Salmonella enterica* strains’ susceptibility to 24 commonly used antimicrobials was assessed using the disc diffusion method. The isolates were MDR, with MAR index ranging from 0.63 to 0.75. All isolates were resistant to AMC, CZ, FOX, TE, and FEB. The MIC ranges were 2 to 32 µg / mL for CIP, 4 to 32 µg / mL for CT, and 32 to 128 µg / mL for TGC (Table 1).

### Efflux pump activity of *Salmonella enterica* strains

Efflux pump activity of MDR *Salmonella* strains was evaluated by assessing the ability of bacteria to extrude ethidium bromide from the cell using the cartwheel method. We recorded the fluorescence exhibited by *Salmonella* strains, which grew as a dense mass along a radial line of TSA plates with increasing concentrations of EtBr. The minimal EtBr concentration and the index of efflux activity for *Salmonella* strains are presented in Table 1. The fluorescence of the examined *Salmonella* strains exceeded that of the control (sensitive *S.* Virchow strain), which exhibited fluorescence at 0.25 µg/mL EtBr. Three TGC-treated strains fluoresced at 2.5 µg/mL, four poultry strains at 2 µg/mL, and five strains at 1.5 µg/mL. One CD-TGC-treated strain fluoresced at 1.5 µg/mL, seven at 0.5 µg/mL, and four at 1 µg/mL.

### Antibiotic resistance genes of *Salmonella* Bredeney strains

Two *Salmonella* Bredeney strains exhibiting high efflux index and high MAR index were selected for WGS. Both strains harbored a similar spectrum of antibiotic resistance determinants (Table 2 and Fig. S1).

**Table 2:** Resistance genes of *Salmonella* Bredeney strains to different antimicrobial agents.

*Resistance genes	Unique resistance genes		Antimicrobial class	Resistance mechanism
	Strain 33 (SRR31606420)	Strain 5 (SRR31606421)		
*rrsD*,* APH(3’)-Ia*,* acrD*,* kdpE*,	*AAC(6’)-Iaa*	*APH(3’)-IIb*	Aminoglycoside	Antibiotic inactivation,
*AAC(6’)-Iy*,* APH(6)-Id*,* APH(3’’)-Ib*,* rrsB*,* rrsC*,* rrnB*,* rrsH*		*16S rRNA*		Antibiotic target alteration, antibiotic efflux
*sul2*,* folP*	ND	ND	Sulfonamide	Antibiotic target alteration
*rpoB*			Rifamycin	
*EF-Tu*			Elfamycin	
	*nfsA*	ND	Nitrofuran	
*gyrB*,* gyrA*,* parE mdtK*,* parC*,* mfd*,* emrB*,* patA*,* mdtH*,* emrA*,* emrR*	ND	ND	Fluoroquinolone	Antibiotic target alteration, antibiotic efflux
*yojI*,* bacA*,* pmrB*,* pmrF*,*16S rRNA*,* arnA*	*pmrC*	ND	Peptide antibiotic	
*tetA*,* tetR*,* tetD*,* rrnB*,* rrsB*	*tetB*	ND	Tetracycline	
*PhoP*	ND	ND	Peptide antibiotic, macrolide	
*acrB*,* sdiA*,* soxR*,* ramR*,* acrA*,* acrR*	ND	ND	Tetracycline, phenicol, rifamycin, penam, glycylcycline, cephalosporin, fluoroquinolone	
*alaS*,* cysB*,* mdtB*,* mdtC*,* mdtA*,	ND	ND	Aminocoumarin	
*murA*,* UhpT*,* GlpT*	*mdtD*	ND	Phosphonic antibiotics	Modification of the antibiotic target *MurA*, antibiotic target alteration
*omp36*,* ompF*,* ompK36*	ND	ND	Penem, penam, cephamycin, cephalosporin, carbapenem, monobactam	Reduced permeability to antibiotic, antibiotic efflux, resistance by absence
*marA*,* soxS*,* ramA*	ND	ND	Tetracycline antibiotic, disinfecting agents and antiseptics, penem, phenicol antibiotic, rifamycin antibiotic, penam, cephamycin, glycylcycline, cephalosporin, carbapenem, monobactam, fluoroquinolone antibiotic	Reduced permeability to antibiotic, antibiotic efflux, antibiotic target alteration
*CMY-57*,* CMY-59*,* CMY-61*,* CMY-43*,* CMY-12*	*CMY-5*,* CMY-7*,* CMY-16*,* CMY-15*,* CMY-28*,* CMY-60*,* CMY-23*,* CMY-6*,* CMY-22*,* CMY-95*,* CMY-108*	*CMY-102*,* CMY-94*,* CMY-56*,* CMY-27*,* CMY-4*,* CMY-99*,* CMY-58*,* CMY-36*	Cephamycin	Antibiotic inactivation
*CMY-2*	ND	ND	Penam, cephamycin, cephalosporin, carbapenem	
*CMY-42*	*CMY-42*	ND	Cephamycin, cephalosporin	
*TEM-116*	*TEM-116*	ND	Penam, penem, cephalosporin, monobactam	
*OXA-115*	*OXA-115*	ND	Penam, carbapenem	
*msbA*	ND	ND	Nitroimidazole	Antibiotic efflux
*cpxA*,* baeR*,* baeS*	ND	ND	Aminocoumarin antibiotic, aminoglycoside antibiotic	
*acrF*,* acrE*	ND	ND	Penam, cephamycin, cephalosporin, fluoroquinolone antibiotic	
*H-NS*	ND	ND	Tetracycline, penam, cephamycin, cephalosporin, fluoroquinolone antibiotic, macrolide antibiotic	
*CRP*,* mdtF*	ND	ND	Penam, fluoroquinolone, macrolide	
*floR*,* emrD*	*mdtL*	ND	Phenicol antibiotic	
*tolC*	ND	ND	Peptide antibiotic, tetracycline, aminoglycoside, disinfecting agents, antiseptics, penem, phenicol, rifamycin, aminocoumarin, penam, cephamycin, glycylcycline cephalosporin, carbapenem, fluoroquinolone, and macrolide	
*mdsC*	*mdsA*	*golS*	Phenicol antibiotic, penem, penam, cephamycin, cephalosporin, carbapenem, monobactam	
*mdtM*	ND	ND	Nucleoside antibiotic, disinfecting agents and antiseptics, phenicol, lincosamide, and fluoroquinolone	
*mdfA*	ND	ND	Phenicol antibiotic, tetracycline	
*cpxR*			Peptide antibiotic, sulfonamide, diaminopyrimidine, aminocoumarin, aminoglycoside, phenicol, tetracycline, penam, cephalosporin, carbapenem, monobactam, fluoroquinolone, macrolide, penem, and cephamycin	
*robA*	ND	ND	Tetracycline, disinfecting agents and antiseptics, phenicol, rifamycin, penam, glycylcycline, cephalosporin, fluoroquinolone, and macrolide	
*mexB*	*nalD*	ND	Peptide antibiotic, sulfonamide, diaminopyrimidine, phenicol, aminocoumarin, tetracycline, penam, cephalosporin, carbapenem, monobactam, fluoroquinolone, macrolide, penem, and cephamycin	
*leuO*	ND	ND	Nucleoside antibiotic, disinfecting agents and antiseptics	
*mexV*	*mexV*	ND	Disinfecting agents and antiseptics, phenicol, tetracycline, macrolide, fluoroquinolone.	
	*oprJ*	ND	phenicol, diaminopyrimidine, aminocoumarin, tetracycline, aminoglycoside antibiotic, penam, cephalosporin, fluoroquinolone antibiotic, and macrolide	
	ND	*smeE*	phenicol, tetracycline, fluoroquinolone, and macrolide	

* Resistance genes that are found in both strains. ND stand for not detected genes.

### Antibiotic efflux genes

Antibiotic efflux *acrB*,* sdiA*,* soxR*,* ramR*,* acrA*, and *acrR* genes mediate tetracycline resistance. Penem, penam, cephalosporin, carbapenem, monobactam resistance is mediated by *omp36*,* ompF*,* ompK36*,* acrB*,* sdiA*,* soxR*,* ramR*,* acrA*,* acrR*,* acrF*,* acrE*,* H-NS*,* CRP*,* mdtF*,* tolC*,* mdsC*,* marA*,* soxS*,* ramA*,* mdtM*,* mdfA*,* cpxR*,* robA*,* mexB*, and *leuO* genes. Fluoroquinolone resistance primarily attributed to antibiotic target alteration and efflux: *gyrB*,* gyrA*,* parE*,* mdtK*,* parC*,* mfd*,* emrB*,* patA*,* mdtH*,* emrA*, and *emrR. msbA*. *cpxA*,* baeR*, and *baeS* efflux genes mediate aminocoumarin resistance. Both *tolC* and *mexB* efflux genes are the cause of peptide antibiotic resistance.

### Antibiotic target alteration

Target alteration *tetA*,* tetR*,* tetD*,* rrnB*, and *rrsB* genes mediate tetracycline resistance. The *rpoB* gene mediates rifamycin resistance. Moreover, phosphonic acid resistance is mediated by antibiotic target alteration genes (*murA*,* UhpT*,* and GlpT). alaS*,* cysB*,* mdtB*,* mdtC*, and *mdtA* genes mediate aminocoumarin resistance. *YojI*,* bacA*,* pmrB*,* pmrF*,* arnA*, and *PhoP* genes mediate peptide antibiotic resistance. *EF-Tu* target alteration gene mediates elfamycin resistance. *Sul2* and *folP* are the main causes of sulfonamide resistance.

### Antibiotic inactivation genes

Aminoglycoside resistance is mediated by antibiotic inactivation through *rrsD*,* APH(3’)-Ia*,* acrD*,* kdpE*,* AAC(6’)-Iy*,* APH(6)-Id*,* APH(3’’)-Ib*,* rrsB*,* rrsC*,* rrnB*, and *rrsH* genes. *CMY-59*,* CMY-61*,* CMY-43*, and *CMY-12* mediate cephamycin resistance.

### Unique genes in S. Bredeney strains of duck and chicken origin

Strain 33 (accession no SRR31606420), isolated from a duck, exhibited unique genes associated with antibiotic resistance. These included genes from the *CMY/CMY-2/CFE/LAT* family (*CMY-16*,* CMY-15*,* CMY-60*,* CMY-6*,* CMY-22*,* CMY-23*,* CMY-5*,* CMY-108*,* CMY-57*,* CMY-95*,* CMY-28*,* CMY-7*,* CMY-42*), which encodes antibiotic inactivation enzymes. Additionally, it contained genes encoding efflux pumps (*FloR* family, *mdtD*, *tetB*, *mdtL*, *mdsA*, *nalD*, *mexV*, and *oprJ*). Furthermore, target alteration genes *nfsA* and *pmrC*, and antibiotic inactivation genes *TEM*-116 and *OXA*-115 were identified (Tables 2 and 3).

**Table 3:** Antimicrobial resistance mechanisms and resistance genes of *Salmonella* Bredeney strains.

AMR mechanism	Resistance genes
Antibiotic inactivation enzyme	*CMY/CMY-2/CFE/LAT family*,* AAC(6’)-Ic*,* f*,*g*,* h*,*j*,* k*,*l*,* r-z*, * APH(3’’)-I*,* APH(3’)-I*,* APH(6)-Ic/APH(6)-Id*
Antibiotic resistance gene cluster, cassette, or operon	*MarA*,* MarB*,* MarR*
Antibiotic target in susceptible species	*Alr*,* Ddl*,* dxr*,* EF-G*,* EF-Tu*,* folA*,* Dfr*,* folP*,* gyrA*,* gyrB*,* inhA*, * fabI*,* Iso-tRNA*,* kasA*,* MurA*,* rho*,* rpoB*,* rpoC*,* S10p*,* S12p*
Antibiotic target protection protein	*BcrC*
Efflux pump conferring antibiotic resistance	*AcrAB-TolC*,* AcrAD-TolC*,* AcrEF-TolC*,* AcrZ*,* EmrAB-TolC*, * EmrD*,* MacA*,* MacB*,* MdfA/Cmr*,* MdtABC-TolC*,* MdtL*, * MdtM*,* MexPQ-OpmE*,* OprM*,* FloR family*,* OprM family*,* SugE*, * Tet(B)*,* TolC/OpmH*
Gene conferring resistance via absence	*gidB*
Protein altering cell wall charge conferring antibiotic resistance	*GdpD*,* PgsA*
Regulator modulating expression of antibiotic resistance genes	*AcrAB-TolC*,* EmrAB-TolC*,* H-NS*,* OxyR*

AMR: antimicrobial resistance mechanism.

Strain 5, isolated from chicken, also exhibited unique resistance genes. These included *APH(*3’)-*IIb* and various CMY family members (*CMY-102*,* CMY-94*,* CMY-56*,* CMY-27*,* CMY- 4*,* CMY-99*,* CMY-58*, and *CMY-36*) encoding antibiotic inactivation enzymes. Efflux pump genes *golS* and *smeE* were also present (Table 2).

### Multilocus sequence typing and plasmid profile

Strain 5 (accession no SRR31606421) shows less than 100% identity to alleles aroC_319, dnaN_63, hemD_303, hisD_16, purE_41, sucA_15, and thrA_3. Based on MLST analysis, its nearest sequence type (ST) is ST 897, indicating the highest similarity. Strain 33 also shows less than 100% identity to alleles aroC_634, dnaN_140, hemD_49, hisD_16, purE_41, sucA_15, and thrA_3. Its nearest STs are 241, 3800, 897, and 8185.

Strain 5 harbors a diverse array of plasmids, including *Col(pHAD28)*, *Col440I*, *ColRNAI*, *IncFII(29)*, *IncFII(Cf)*, *IncFII(S)*, *IncFII(Yp)*, *IncFII(p96A)*, *IncFII(pAMA1167-NDM-5)*, *IncFII(pRSB107)*, *IncFII(pSE11)*, *IncFII(pSFO)*, *pSL483*, and *pXuzhou21*. Strain 33 contains plasmids *IncC*, *IncFII(p96A)*, and *pSL483*.

### Transmission electron microscopy of tigecycline-loaded chitosan/dextran sulfate nanocapsules

**Fig. 2:**
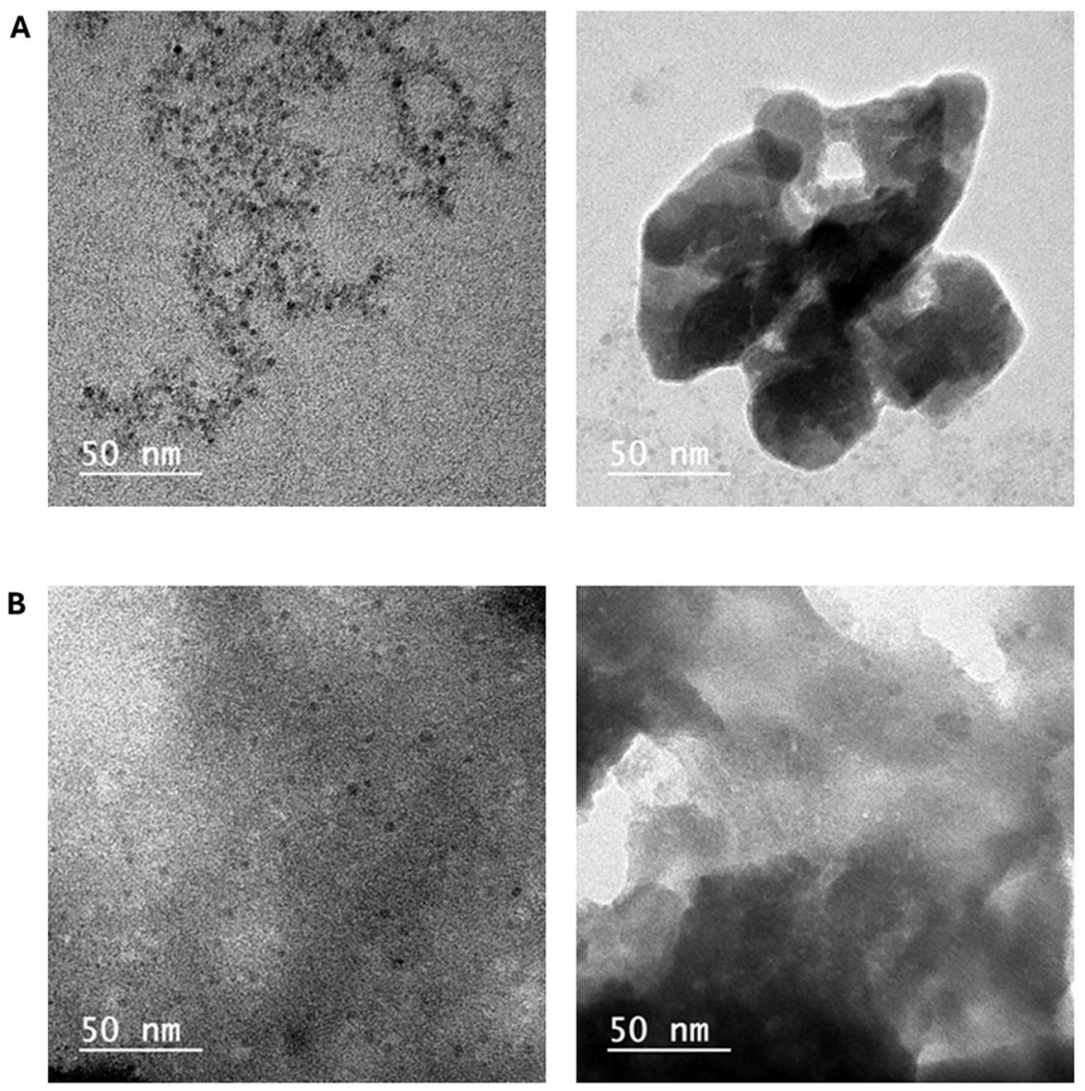
A: Chitosan dextran sulfate nanoparticles and B: tigecycline-encapsulated chitosan dextran sulfate nanoparticles.

The size and morphology of the produced nanoparticles were assessed using TEM (Fig. 2). The blank nano-chitosan/dextran sulfate (Fig. 2A) exhibits a semi-spherical shape with a nodal structure and an average size of 50 to 80 nm. The TGC-loaded CD nanocapsules have a spherical morphology with an average size ranging from 34 to 75 nm (Fig. 2B).

### Particle size distribution, polydispersity index, and zeta potential

**Fig. 3:**
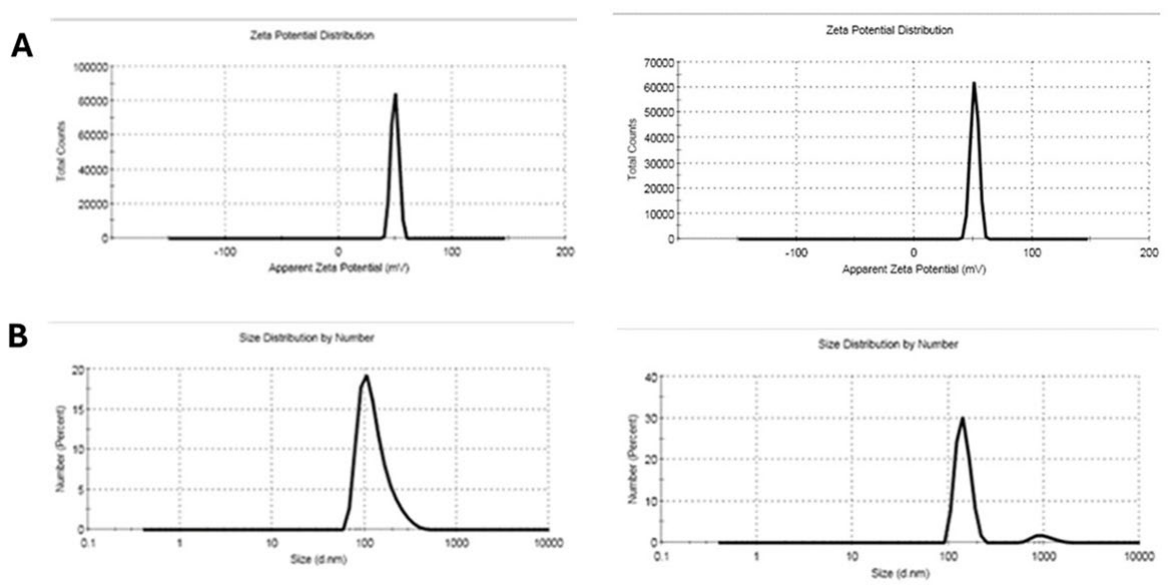
Zeta potential and particle size of chitosan dextran sulfate nanoparticles encapsulated tigecycline and unloaded chitosan dextran sulfate nanoparticles (A). Mean particle size was 129.4 with tigecycline (A) and 144.4 without drug tigecycline (B).

Particle size was assessed using dynamic light scattering (DLS), which measures the Brownian motion of nanoparticles. Figure 3A and B show the average particle sizes of chitosan/dextran sulfate nanoparticles: 129.4 nm with TGC and 144 nm without. Figure 3 also displays the zeta potential of the synthesized nanoparticles. Upon TGC encapsulation, the zeta potential changed. The zeta potential was 49.4 mV for nanoparticles with TGC and 51.3 mV for nanoparticles without TGC. These values indicate highly stable chitosan/dextran sulfate nanoparticles.

### X-ray diffraction (XRD)

**Fig. 4:**
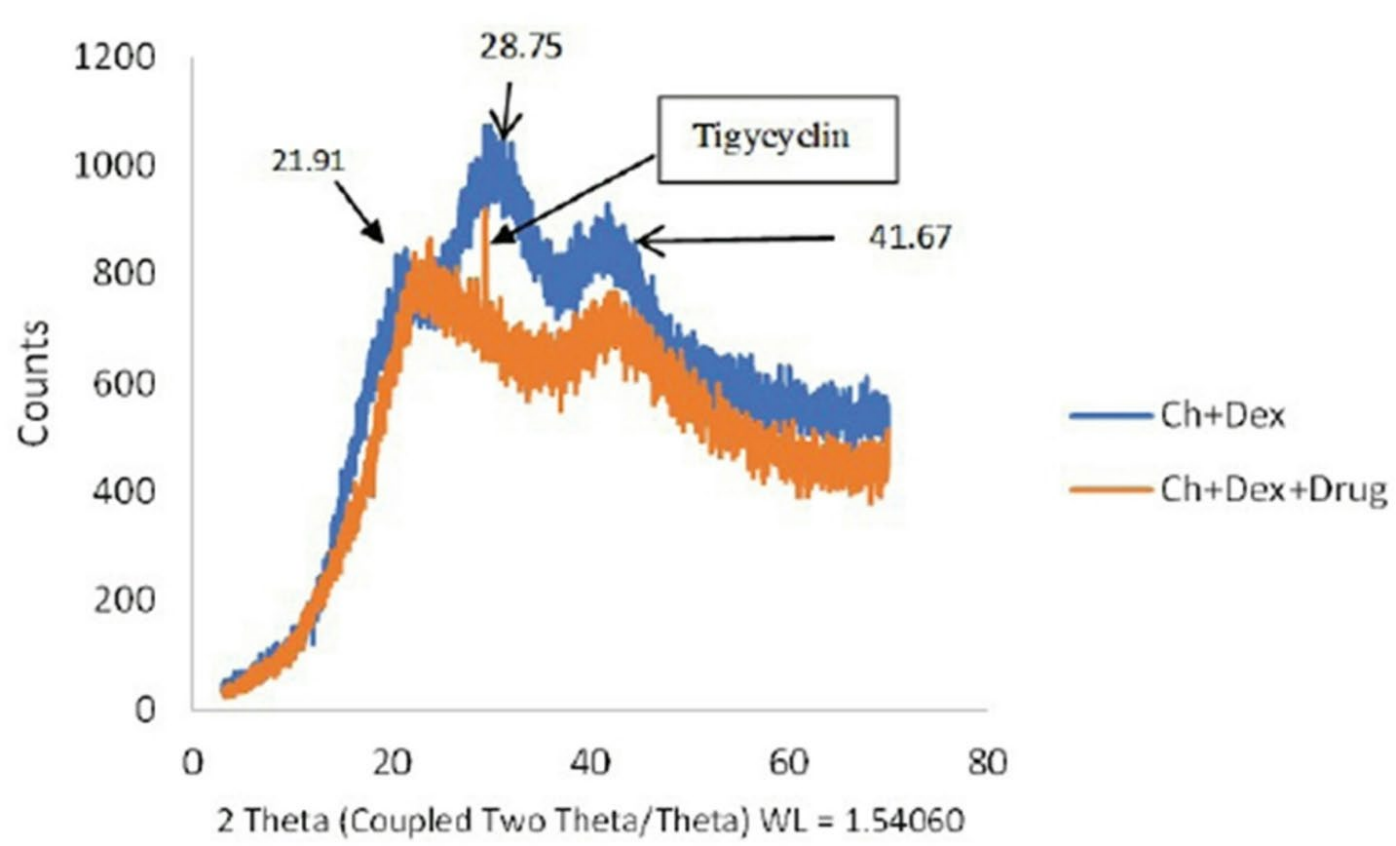
X-ray diffraction (XRD) of chitosan nanoparticles without drug and chitosan nanoparticles with tigycyclin.

Figure 4 shows the XRD patterns of chitosan/dextran sulfate nanoparticles with and without tigecycline. The XRD pattern of the drug-loaded nanoparticles shows a new sharp peak at 2θ: 29.39 Ǻ, which is consistent with the presence of tigecycline. The chitosan/dextran sulfate nanoparticles (without drug) exhibit characteristic diffraction peaks (Fig. 4). The reduced intensity of these peaks in the TGC-loaded sample suggests an interaction between the TGC and the nanoparticle matrix. The absence of additional peaks indicates the relative purity of the nanoparticles.

### Kinetic release of tigecycline from chitosan dextran sulphate nano-capsule

**Fig. 5:**
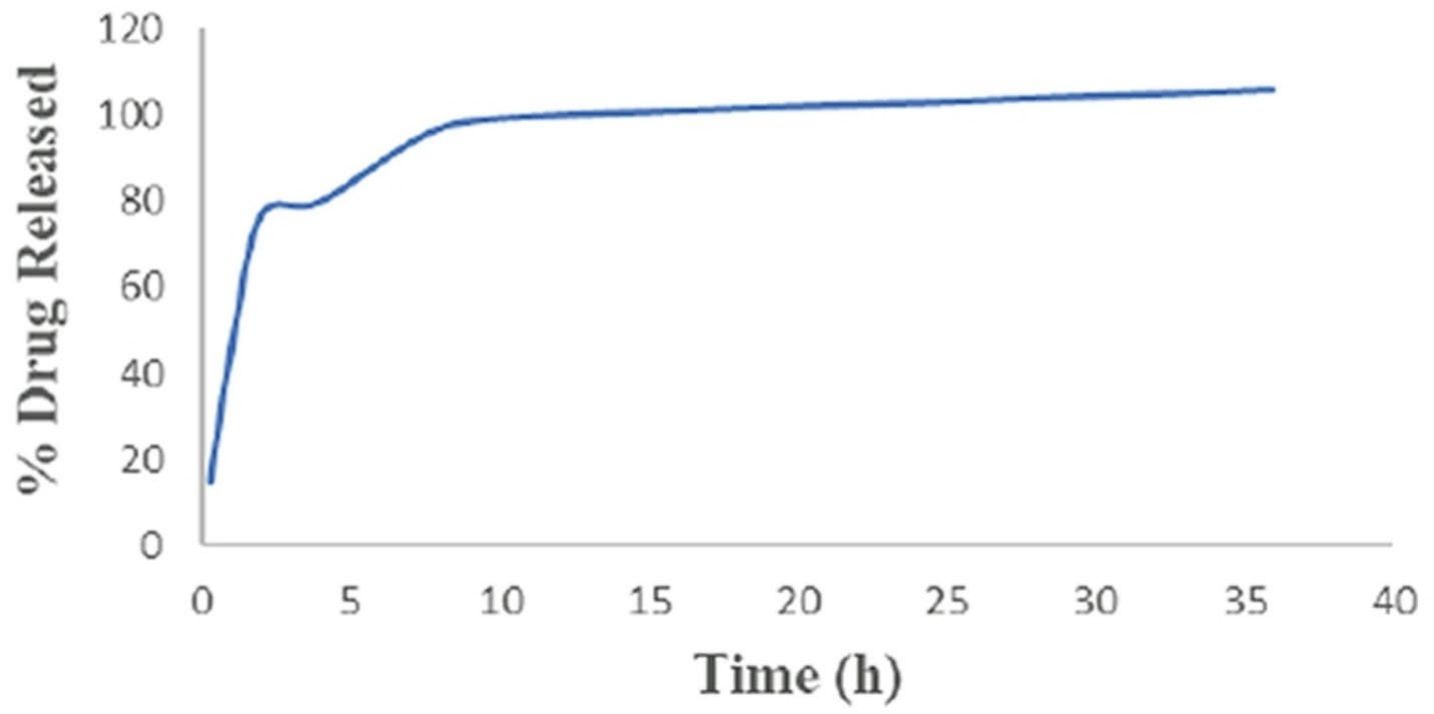
Release profile of tigecycline from chitosan dextran sulphate nano-encapsulation in phosphate-buffered saline solution at pH 7.4.

Table 4; Fig. 5 summarize the in vitro release profile of nano-encapsulated TGC in PBS (pH 7.4), simulating physiological conditions, over 36 h. A small but noticeable initial burst release of TGC was observed within the first 2 h, reaching 0.462 mg (representing 77% of the total loaded drug). This burst likely represents drug adsorbed on or near the surface of the nanocarriers. After this initial phase, the release rate significantly decelerated, with 100% of the drug being released by 8 h. The complete release of the encapsulated drug within this timeframe suggests that the nanocapsules remained intact and fully discharged their payload under these conditions.

**Table 4:** Stability and kinetic release profile of tigecycline from chitosan dextran sulphate nano-capsule.

Time	Concentration	Cumulative release (mg)	Tigecycline release %
30 min.	0.003	0.090	0.15
1 h.	0.009	0.273	0.46
2 h.	0.015	0.462	0.77
4 h.	0.015	0.477	0.80
8 h.	0.018	0.582	0.97
12 h.	0.018	0.600	100
24 h	0.018	0.618	103
36 h.	0.018	0.636	106

### Tigecycline loading capacity and efficiency

After encapsulation, the concentration of tigecycline in the supernatant was analyzed by UV-Vis spectroscopy to determine the amount of unencapsulated drug, which was found to be 0.0042 mg. The total amount of loaded drug was 15.9958 mg, resulting in a Drug Loading Efficiency (DLE) of approximately 99.97%. These results demonstrate a highly efficient encapsulation of tigecycline, particularly highlighting the effectiveness of the formulation utilizing chitosan and dextran sulfate.

### Drug release kinetics analysis

Different kinetic models, including zero-order, first-order, Higuchi, and Korsmeyer–Peppas, were applied to analyze the in vitro release profile of the TGC-loaded CD NPs. The Korsmeyer–Peppas model showed the best fit to the TGC release data, particularly for the early phase of release (up to 2 h) with a correlation coefficient (R2 = 0.9574). The calculated release exponent (n) was 1.161, which indicates a Super Case II transport mechanism (polymer relaxation–controlled release) (Table 5). This suggests that the release process is primarily controlled by polymer matrix relaxation and erosion, rather than by diffusion alone. This profile is characteristic of crosslinked or highly swollen polymeric systems, underscoring the significant influence of the chitosan-dextran sulfate matrix on the drug release kinetics over time.

**Table 5:** Analysis of tigecycline kinetics release.

Model	R2R^2R2 (Goodness of Fit)
Zero-order	0.3563
First-order	0.2586
Higuchi	0.5421
Korsmeyer–Peppas	0.9574

The Higuchi model showed a moderate fit, indicating that diffusion might be a contributing factor over time. Conversely, the Zero- and First-order models exhibited poor fits, suggesting that the release is not simply concentration-dependent or governed by a constant rate.

### Safety of TGC-loaded nano-encapsulation

To assess the biocompatibility of CD-TGC nanocapsules, a cytotoxicity assay was conducted on the BJ normal human fibroblast cell line using both TGC-loaded and blank (unloaded) chitosan-dextran sulfate nanocapsules. The results showed that the TGC-loaded nanocapsules exhibited low cytotoxicity, with 12.60% cell inhibition at 100 ppm. In contrast, the blank nanocapsules demonstrated a -25.79% cytotoxicity, indicating enhanced cell proliferation. DMSO, used as a vehicle control, showed mild cytotoxicity (5%), while the negative control showed no cytotoxic effect (0%). Due to the minimal observed cytotoxicity, no IC₅₀ or IC₉₀ values could be determined.

### MIC values of TGC-loaded CD NPs

Table 2 shows the MIC values of TGC and CD-TGC against *Salmonella enterica* strains. CD-TGC was more effective, with MICs of 0.5–1 µg/mL. TGC showed limited antibacterial activities, with high MIC values ranging from 32 to 128 µg / mL. CD-TGC decreased MIC 7-fold, from 128 to 1 µg/mL, in one *S.* Typhimurium strain. In the other 11 strains, CD-TGC reduced the TGC MIC 6-fold, from 64 to 1 µg/mL (*n* = 6) and from 32 to 0.5 µg/mL (*n* = 5).

### Time-kill kinetics

**Fig. 6:**
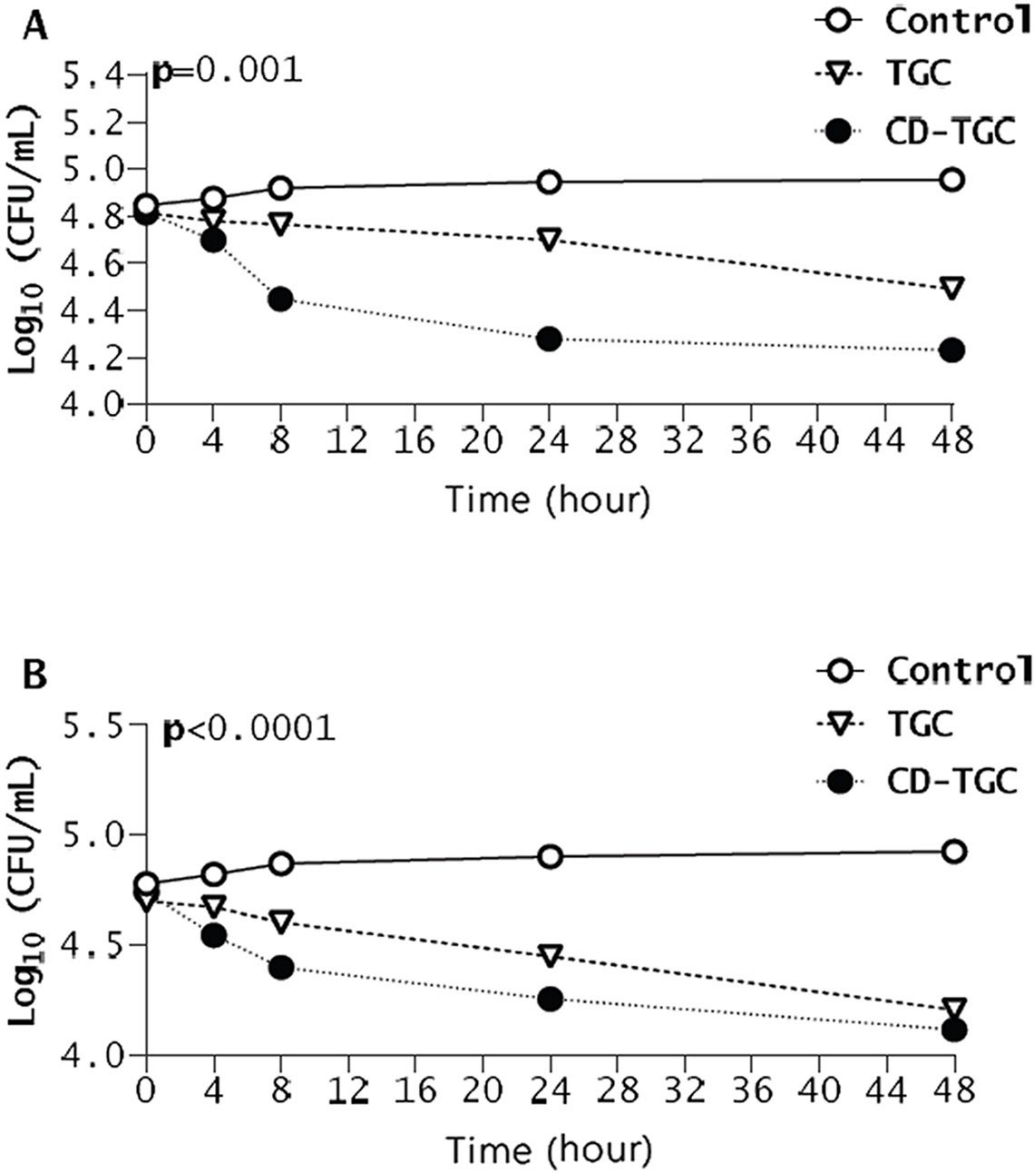
Time-kill curves of *S.* Typhimurium strain with two inoculums of 8 × $\:{10}^{6}$ CFU/ml (A) and 8 × $\:{10}^{4}$ CFU/ml (B) incubated with 2 µg/mL tigecycline (TGC) and 2 µg/mL tigecycline-loaded chitosan-dextran sulfate (CD-TGC) nanocapsule.

With an initial inoculum of 8 × 10^6^ CFU/mL, the colony count in CD-TGC treatment significantly decreased to 4.23 CFU/mL at 48 h (*p* = 0.001) (Fig. 6A). At a lower initial inoculum of 8 × 10^4^ CFU/mL, the bacterial count decreased to 4.11 CFU/mL (Fig. 6B), indicating a strong antibacterial effect of CD-TGC.

### Tigecycline–loaded Chitosan dextran sulfate nanocapsules downregulate efflux pump genes

**Fig. 7:**
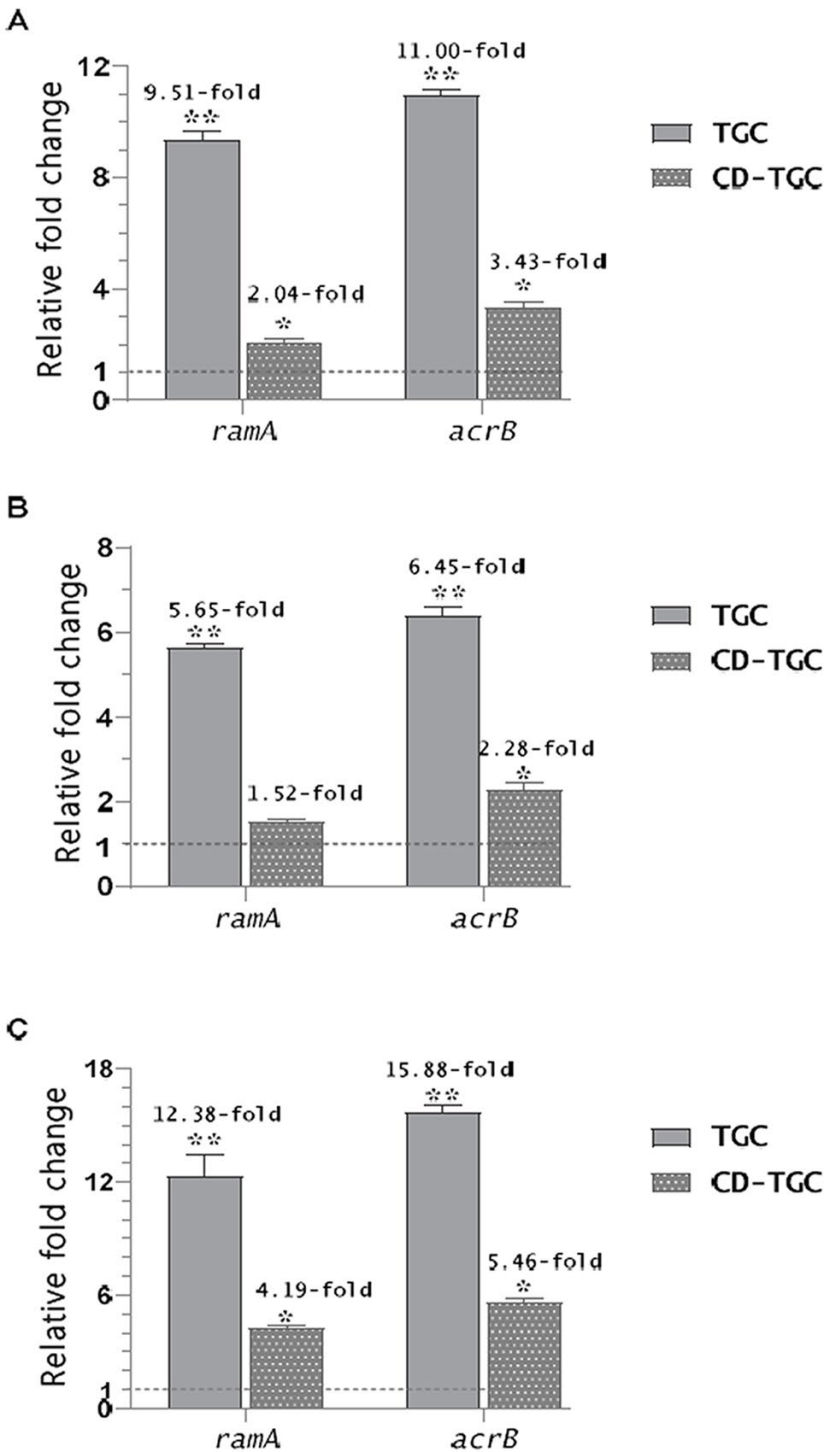
Relative fold change of *ramA* and *acrB* genes in *S.* Typhimurium (A), *S*. Magheroftt (B), and *S.* Bredeney (C) upon treatment with TGC MIC and CD-TGC MIC. The relative mRNA expression level of genes was quantified compared to expression levels in the untreated strains. * *p* < 0.05; ** *p* < 0.01 differ significantly with the control according to Student’s t-test.

The CD-TGC treated strains showed significant downregulation of *ram*A, with fold changes of 2.04 in strain 1, 1.52 in strain 2, and 4.19 in strain 3. Similarly, *acr*B expression was reduced by 3.43-fold in strain 1, 2.28-fold in strain 2, and 5.46-fold in strain 3 compared to untreated strains (*p* < 0.05). Significant differences in the expression of these two genes were detected between the CD-TGC-treated and untreated strains, except for the *ram*A gene in strains 1 and 2 (Fig. 7).

### In vivo efficacy of tigecycline–loaded Chitosan dextran sulfate nanocapsules

**Fig. 8:**
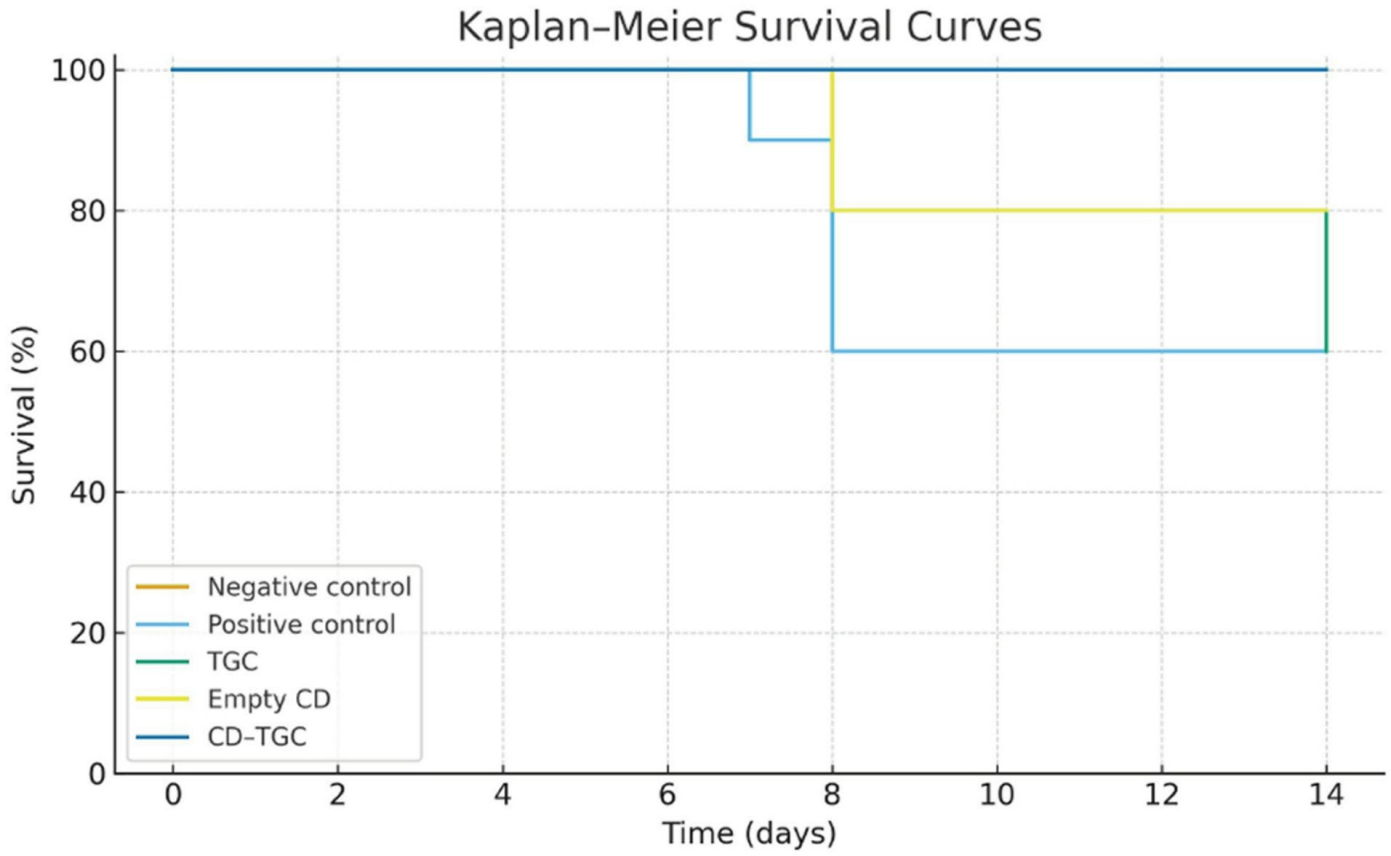
Survival rates of mice (*n* = 10 in each group) that were infected with *S.* Typhimurium and treated with TGC, unloaded CD NPs, and CD-TGC.

Mice in the infected, untreated positive control group exhibited lethargy, loss of appetite, weight loss, and diarrhea (with or without mucus) during the experimental period. All mice in this group died within 8 days of infection (Fig. 8). The CD-TGC treatment group had a 100% survival rate, followed by the unloaded CD NPs group (80%). The TGC treatment group experienced a higher mortality rate, with a 40% survival rate.

### Effect of different treatments on hemato-biochemical parameters

Compared to the negative control group, the positive control group showed significant increases (*p* < 0.05) in ALT, AST, ALP, LDH, creatinine, and urea levels, and marked decreases (*p* < 0.05) in TP and albumin Alb levels. Compared to the untreated control group, all treatments (TGC, unloaded CD NPs, and CD-TGC) significantly decreased (*p* < 0.05) serum levels of ALT, AST, ALP, LDH, creatinine, and urea in *S. Typhimurium*-infected mice. However, CD-TGC treatment resulted in the most substantial reductions (*p* < 0.05) in these markers, with levels approaching normal ranges. The treatment demonstrated significant effects on TP and Alb, with notable increases observed in the CD-TGC group, followed by the unloaded CD NPs -treated group, and then the TGC group.

The Hb concentration in the positive control group was significantly reduced (*p* < 0.05) compared to the negative control group. In contrast, Hb levels were substantially increased in all treatment groups, with the highest concentration in CD-TGC, followed by unloaded CD NPs, and TGC. Total leukocyte count (WBCs), lymphocytes, and neutrophils were considerably higher in the positive control group. The CD-TGC-treated group also showed a substantial increase in WBCs, lymphocytes, and neutrophils (Table 6).

**Table 6:** Effect of different treatments on blood biochemical and hematological attributes of different mice groups.

Items	NC	PC	TGC	Unloaded CD NPs	CD-TGC	*p*-value
Blood protein						
TP g/dl	6.97 ± 0.01^a^	5.14 ± 0.01^c^	6.50 ± 0.01^b^	6.57 ± 0.01^ab^	6.63 ± 0.01^ab^	0.0027
Alb g/dl	3.95 ± 0.02^a^	3.28 ± 0.04^c^	3.65 ± 0.03^b^	3.74 ± 0.06^ab^	3.83 ± 0.03^ab^	0.0013
Liver function						
ALT U/L	57.17 ± 1.42^c^	74.56 ± 2.18^a^	63.39 ± 2.30^b^	60.11 ± 1.79^b^	58.80 ± 2.44^b^	0.0001
AST U/L	172.16 ± 4.83^b^	208.73 ± 6.13^a^	187.62 ± 5.69^c^	184.36 ± 5.57^c^	180.06 ± 6.83^bc^	0.0008
ALP U/L	111.3 8 ± 1.55^c^	147.12 ± 4.66^a^	123.13 ± 3.67^b^	118.67 ± 2.76^b^	113.13 ± 3.25^bc^	0.0001
LDH U/L	373.16 ± 2.53^c^	395.62 ± 5.41^a^	391.56 ± 4.32^ab^	385.07 ± 2.63^ab^	382.67 ± 5.62^bc^	0.0047
Total bilirunin	0.18 ± 0.01^c^	0.38 ± 0.04^a^	0.22 ± 0.01^b^	0.20 ± 0.01^bc^	0.19 ± 0.00^c^	0.0013
Kidney function						
Creatinine mg/dL	0.81 ± 0.01^cd^	0.87 ± 0.01^a^	0.86 ± 0.01^ab^	0.85 ± 0.01^bc^	0.80 ± 0.01^d^	0.0001
Urea mg/dL	34.18 ± 1.93^bc^	47.86 ± 1.44^a^	38.56 ± 0.63^b^	37.78 ± 0.87^bc^	33.99 ± 1.25^c^	<0.0001
Uric acid mg /dl	3.62 ± 0.07^c^	5.63 ± 0.14^a^	5.37 ± 0.01^a^	4.40 ± 0.08^b^	3.82 ± 0.15^c^	<0.0001
Hematological attributes						
Hb g/dl	14.69 ± 0.30^a^	12.83 ± 0.17^c^	14.00 ± 0.08^b^	14.18 ± 0.29^ab^	14.51 ± 0.22^ab^	<0.0001
WBCs (10^3^/µl)	9.18 ± 0.37^cd^	12.06 ± 0.31^a^	10.69 ± 0.32^b^	10.14 ± 0.16^bc^	8.56 ± 0.35^d^	0.0001
Neutrophil %	25.33 ± 1.45^c^	38.67 ± 0.88^a^	32.83 ± 0.73^b^	31.67 ± 0.88^b^	23.00 ± 1.15^c^	<0.0001
Lymphocytes %	62.67 ± 0.88^c^	69.67 ± 0.88^a^	68.33 ± 0.88^ab^	66.00 ± 0.58^b^	60.33 ± 0.88^c^	<0.0001

Data are presented as least square means ± standard errors. ALT, alanine aminotransferase; AST, aspartate aminotransferase; ALP, alkaline phosphatase; LDH, lactate dehydrogenase; TP, total protein; Alb, albumin; Hb, hemoglobin; WBCs, white blood cell counts. ^a, b,c^Means within a row without a common superscript letter differ at *p* < 0.05.

### Effect of tigecycline-loaded chitosan dextran sulfate nanocapsules on *S.* Typhimurium burden

**Fig. 9:**
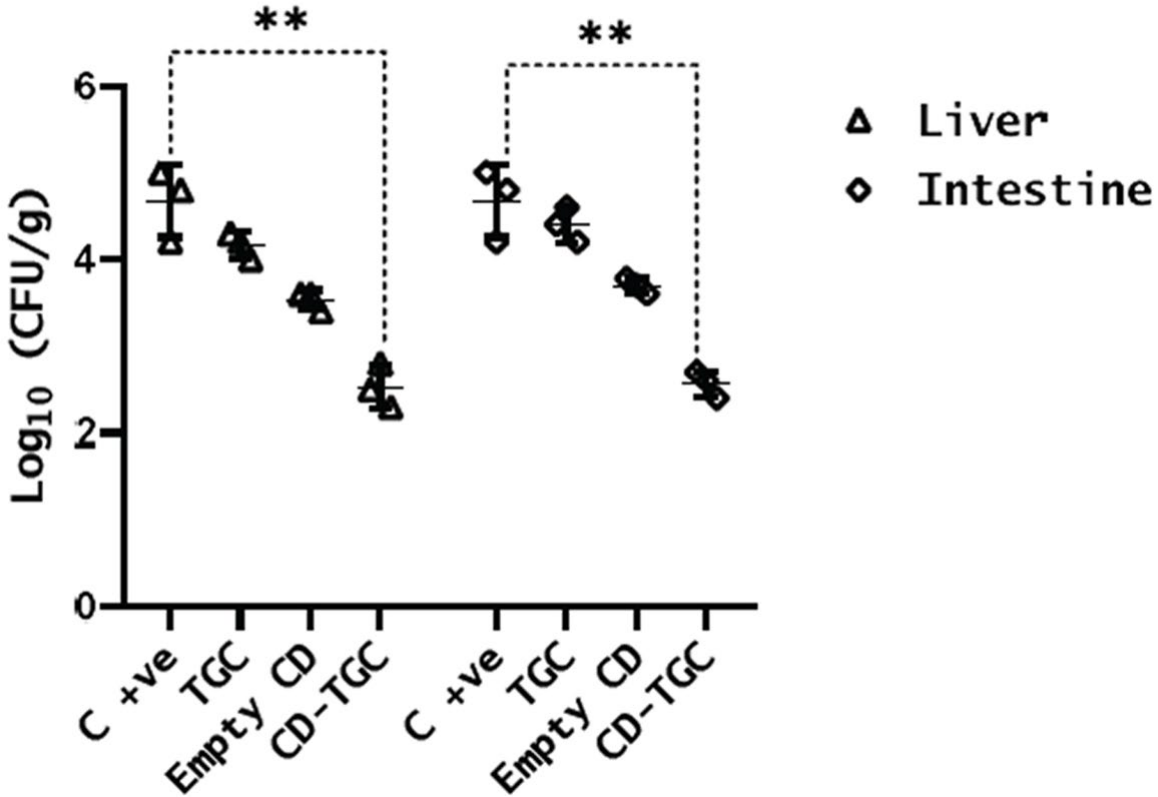
Total *S.* Typhimurium counts in the liver and intestine of mice after four days of treatment with TGC, unloaded CD NPs, and CD-TGC. ** *p* < 0.001 was calculated by one-way ANOVA.

The presence of *Salmonella* in the organs of mice was determined by inoculating the organ lysates onto XLD agar media. All treated groups showed significant decreases (*p* < 0.001) in *S.* Typhimurium burden in the liver and intestine compared to the positive control group, with the CD-TGC group exhibiting the lowest bacterial burden (Fig. 9).

### Effect of different treatments on the histology of the liver and intestine

**Fig. 10:**
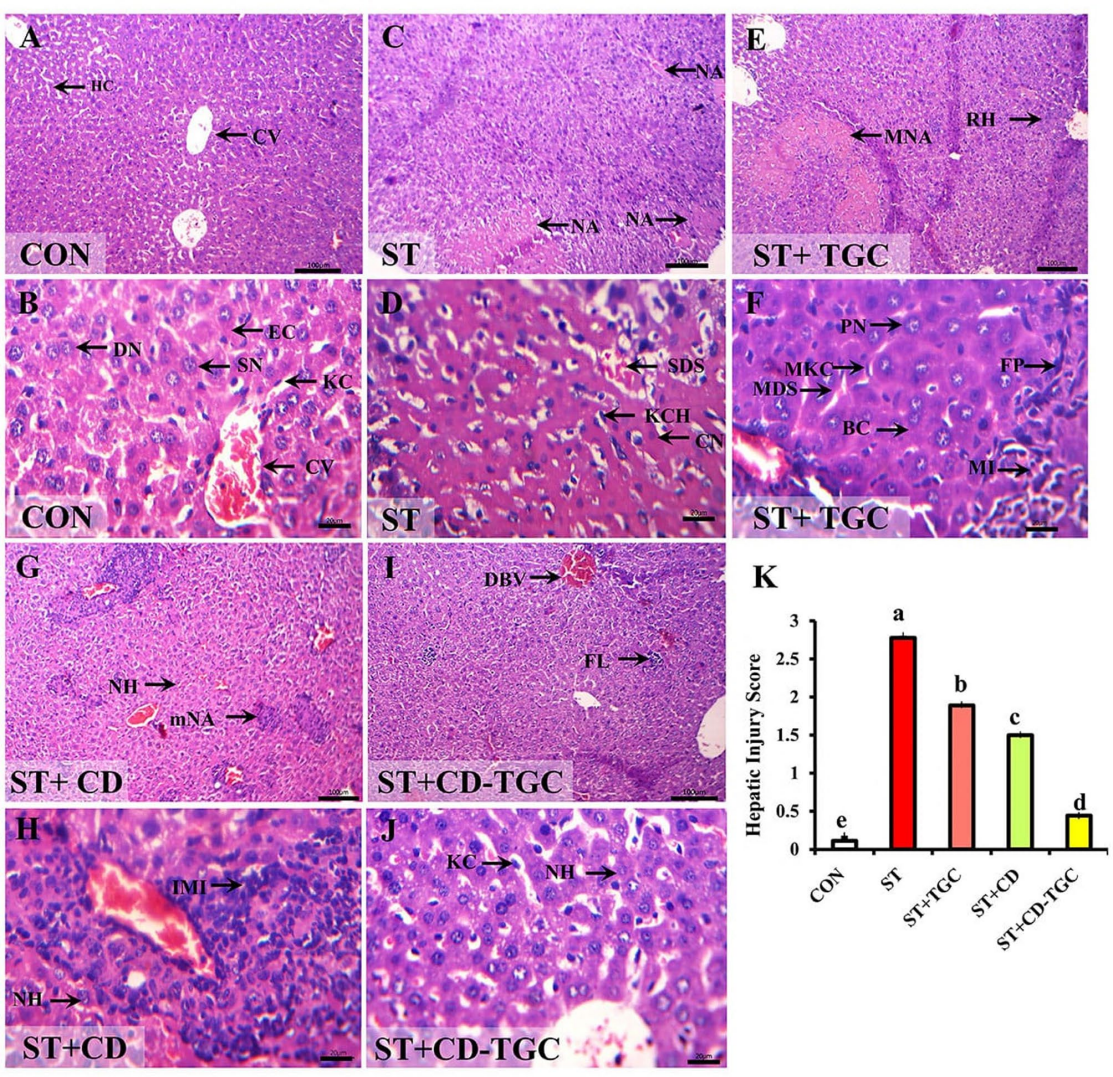
TGC and/or unloaded CD NPs ameliorated the *S.* Typhimurium infected group induced histopathological alterations in mice’s liver tissues. Representative photomicrographs of the H&E-stained hepatic tissue sections showing the control (A), *S.*Typhimurium infected group (C), TGC (E), unloaded CD NPs (G), CD-TGC groups (I) and their respective higher magnifications (B, D, F, H, and J). A, B: control group displaying normal central vein (CV), hepatic cords (HC) with hepatocytes of eosinophilic granular cytoplasm (EC), rounded central single (SN) or double vesicular nuclei (DN), and kupffer cells (KC). C, D: *S.* Typhimurium infected group demonstrating multiple areas of variable-sized necrotic areas (NA) of coagulative necrosis (CN), severely dilated and congested sinusoid (SDS) with Kupffer cell hyperplasia (KCH). E, F: TGC group displaying moderately sized necrotic areas (MNA), moderately dilated and congested sinusoids (MDS), moderately hyperplastic Kupffer’s cells (MKC), interstitial mononuclear cell infiltration (MI), fibroblast proliferation (FP), and regenerated hepatocytes of stippling basophilic cytoplasm (BC) and pale nuclei (PN). G, H: unloaded CD NPs group showing a few scattered minute necrotic areas (mNA), intense mononuclear cell infiltration (IMI) around the portal area, and apparently normal hepatocytes (NH). I, J: CD-TGC group showed normal hepatocytes (NH), few dilated blood vessels (DBV), and a few interstitial lymphocytic aggregates (FL). Scale bars = 100 μm in A, C, E, G, I; and = 20 μm in B, D, F, H, and J. K: Bar charts demonstrate the statistical analysis of the comparative quantification of the hepatic injury scores in all studied groups. Bars carrying different superscript letters (a, b, c, d, and e) are significantly different as analyzed by the one-way ANOVA test, followed by the multiple comparisons by Duncan’s Post-hoc test (*p* < 0.05). Values are the mean of 6 mice per group ± S.E.M.

The H&E-stained sections of the liver tissues of the control-negative group reflected a normal histological structure of hepatic parenchyma (Fig. 10A and B). *S.* Typhimurium-infected group exhibited multiple areas of variable-sized coagulative necrosis that were randomly distributed within the hepatic parenchyma. Severe sinusoidal dilation and congestion with Kupffer cell hyperplasia were characteristic manifestations of *Salmonella* hepatitis (Fig. 10C and D). The TGC group revealed moderately sized necrotic areas, moderately dilated and congested sinusoids with moderately hyperplastic Kupffer`s cells beside interstitial mononuclear cell aggregates with fibroblast proliferation. Regenerated hepatocytes with stippling basophilic cytoplasm and pale nuclei reflected the regenerative process (Fig. 10E and F). The unloaded CD NPs group exhibited a few randomly scattered minute necrotic areas with highly mononuclear cell infiltration around the portal areas. Normal hepatic parenchyma was common (Fig. 10G and H). CD-TGC restored normal hepatic histomorphologic features except for a few dilated blood vessels and a few interstitial lymphocytic aggregates. Sometimes, hepatic cells showed minute vacuoles within their cytoplasm (reversible change) (Fig. 10I and J).

**Fig. 11:**
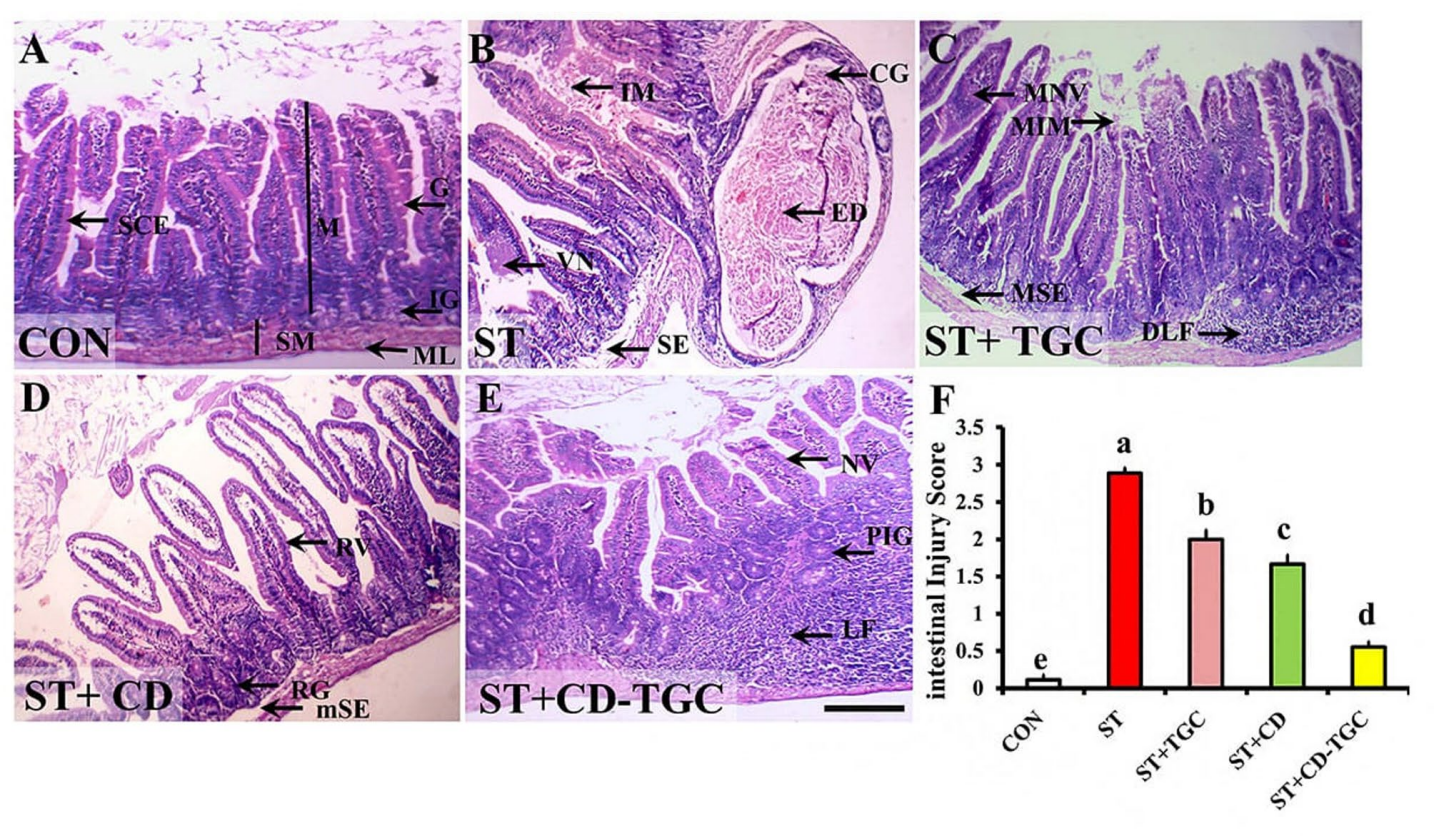
TGC and/or unloaded CD NPs ameliorated the *S.* Typhimurium infected group induced histopathological alterations in mice’s small intestines. Representative photomicrographs of the H&E-stained intestinal tissue sections showing the control (A), *S.* Typhimurium infected group (B), TGC (C), unloaded CD NPs (D), and CD-TGC groups (E). A: control group displaying normal intestinal mucosa (M) of simple columnar absorptive epithelium (SCE) with interspersed goblet cells (G) covering villi, intestinal glands (IG), and submucosa (SM), muscularis (ML). B: *S.* Typhimurium infected group demonstrating villus necrosis (VN), intervillous stuck mucous (IM), cystic intestinal glands (CG) containing eosinophilic debris (ED), and submucosal edema (SE). C: TGC group displaying moderate necrosis of villus (MNV) with moderately intervillus-stuck mucous (MIM), moderately submucosal edema (MSE), and depleted lymphoid follicles (DLF). D: Unloaded CD NPs group shows regenerated intestinal villi (RV), glands (RG), and mild submucosal edema (mSE). E: CD-TGC group showing normal villi (NV), overcrowded proliferative intestinal glands (PIG), and well-defined submucosal lymphoid follicles (LF). Scale bars in A, B, C, D, and E = 50 μm. F: Bar charts demonstrate the statistical analysis of the comparative quantification of the intestinal injury scores in all studied groups. Bars with distinct superscript letters (a, b, c, d, and e) exhibit significant differences, as determined by the one-way ANOVA test, followed by Duncan’s Post-hoc multiple comparisons (*p* < 0.05). The values represent the mean of six mice per group ± S.E.M.

Semiquantitative assessment showed that hepatic injury scores were significantly higher (*p* < 0.05) in the *S.* Typhimurium-infected (control positive) group (2.78 ± 0.03) compared to the control negative group (0.11 ± 0.07). Treatment with CD-TGC, TGC, or unloaded CD NPs significantly reduced (*p* < 0.05) hepatic injury scores relative to the positive control group. Among these, CD-TGC 0.44 ± 0.001) was the most effective, outperforming TGC (1.89 ± 0.01) and unloaded CD NPs (1.5 ± 0.01) (Fig. 10K; Table 7). The villi were mucosal projections of simple columnar absorptive epithelium with interspersed goblet cells. The intestinal glands (crypts of Lieberkühn) were found between the bases of the villi and the lamina muscularis mucosa (Fig. 11A). However, *S.* Typhimurium-infected group showed necrosis of some villi with mucus stuck in intervillus spaces. Besides, a few cystic intestinal glands were bulged and contained eosinophilic debris. The submucosal edema was also detected (Fig. 11B).

**Table 7:** Hepatic and intestinal lesion scoring among experimental groups infected with *S.* Typhimurium.

Parameter Group	Hepatic injury score	Intestinal injury score
Control	0.11 ± 0.07	0.11 ± 0.07
*S.* Typhimurium infected group	2.78 ± 0.03	2.89 ± 0.07
TGC	1.89 ± 0.01	2 ± 0.12
Unloaded CD NPs	1.5 ± 0.01	1.67 ± 0.12
CD-TGC	0.44 ± 0.001	0.56 ± 0.07

The TGC group revealed moderately necrotic areas in some villi with moderately stuck mucus in intervillous spaces. The submucosa exhibited depletion of some lymphoid follicles and moderate edema (Fig. 11C). In the unloaded CD NPs group, the small intestine revealed normal intestinal mucosa with regenerated intestinal glands and mild submucosal edema (Fig. 11D). The CD-TGC group exhibited normal intestinal villi and overcrowded proliferative intestinal glands. The well-defined submucosal lymphoid follicles were also noticed (Fig. 11E). Semiquantitative assessment of intestinal injury scores significantly increased (*p* < 0.05) in the infected control group (2.89 ± 0.07) compared to the uninfected control group (0.11 ± 0.07). CD-TGC, TGC, and unloaded CD NPs all significantly reduced (*p* < 0.05) these scores compared to the infected control. CD-TGC significantly reduced intestinal injury scores (0.56 ± 0.07) compared to either TGC (2 ± 0.12) or unloaded CD NPs (1.67 ± 0.12) (Fig. 11F).

## Discussion

*Salmonella* is one of the primary zoonotic pathogens included in foodborne epidemics worldwide. *S.* Typhimurium is one of the most recurrent and virulent serovars, representing a considerable global danger to food safety and public health, attributed to the rise of MDR bacteria^48^. As a rare human pathogen linked to sporadic outbreaks, *S. enterica* serotype Bredeney is well known for being isolated from poultry, other animals, and the environment^49^. It has emerged as the third most prevalent serotype in recent years among isolates from human infections that were sent to Ireland’s National *Salmonella* Reference Laboratory for diagnosis. Currently, 87% of *S. enterica* serotype Bredeney isolates from non-human sources and from unrelated human illnesses in Ireland belong to a closely linked group, as established by DNA amplification fingerprinting and pulsed field gel electrophoresis^50^.

*Salmonella* acquires multidrug resistance via efflux pumps that expel antibiotics from bacterial cells, thereby reducing their concentration to non-toxic levels and inactivating them through bacterial enzymes that modify or degrade their structure^51^. The efflux pumps in bacteria facilitate the expulsion of drugs from cells and are also implicated in bacterial stress response, pathogenicity, biofilm formation, and modification of host physiology^52^. Efflux pumps in *Salmonella* are essential for mitigating the adverse effects of bile salts and host defense mechanisms^53^. The detected efflux pump genes conferring antibiotic resistance in *S.* Bredeney strains are *AcrAB-TolC*,* AcrAD-TolC*,* AcrEF-TolC*,* AcrZ*,* EmrAB-TolC*,* EmrD*,* FloR* family, *MacA*,* MacB*,* MdfA/Cmr*,* MdtABC-TolC*,* MdtL*,* MdtM*,* MexPQ-OpmE*,* OprM/OprM* family, *SugE*,* Tet(B)*, and *TolC/OpmH*. In *Salmonella*, the resistance-nodulation-division (RND) proteins are situated in the inner membrane. The outer membrane channel, aided by other proteins, is integral to the drug efflux system, in conjunction with the tripartite system (*AcrAB-ToIC*). It transports substrates such as antibiotics, dyes, detergents, and host metabolites. The absence of RND protein components renders *Salmonella* susceptible to antibiotics, but the overexpression of these genes leads to multidrug resistance^54^. Consequently, research on these proteins is becoming increasingly significant in combating multidrug resistance and reducing the pathogenicity of *Salmonella*^55^. *Ram*A overexpression is crucial for *acr*AB overexpression and promotes efflux-mediated co-resistance to CIP/TGC without prior exposure to TGC^8^. TGC is the ultimate broad-spectrum drug for the treatment of Salmonella infections^56^. Mechanisms encoded by chromosomes or accessory genes contribute to TGC resistance. The horizontal transfer of mobile genetic elements carrying multiple resistance genes may cause the Tet proteins (e.g., *Tet(X)*,* Tet(A)*,* Tet(K)*, and *Tet(M)*) to acquire mutations that result in a decreased susceptibility (i.e., increased MICs) to TGC^57^. Gram-negative bacteria’s resistance to tigecycline is largely due to chromosomally encoded overexpression of resistance-nodulation division (RND) efflux pumps, including *AdeABC*,* AdeFGH*,* AdeIJK*,* MexXY*, and *AcrAB*^52^.

NPs drug delivery Innovative systems are being investigated to address the problems associated with drug use, which include inadequate biodistribution, restricted efficacy, adverse side effects, and insufficient selectivity^58^. It can surmount these restrictions by delivering the drug to the site of action, while safeguarding against fast degradation or clearance. It also increases drug concentration in target tissues, hence allowing for lower doses to mitigate hazardous side effects^59^. There is increased interest in natural-based NPs with antibacterial activities concurrently with antibiotic treatment, as the dose of antibiotics can be reduced and the undesirable effects of drugs can be further minimized^55^. Although previous studies reported that CH has antimicrobial activity^60^. However, it can’t overcome intracellular *S.* Typhimurium infection^61^. So, it must combine with another carbohydrate polymer, DS, to deliver the conjugated drug into the intracellular *Salmonella*^62^.

Our study points to reviving TGC activity to combat MDR *Salmonella* using natural CD capsules to overcome intracellular *Salmonella* infection. To our knowledge, no data have been published on using CD-TGC as an antimicrobial agent against MDR *Salmonella enterica* strains. We used TEM to characterize the nanoparticles, which showed CD-TGC, a semi-spherical shape, and a slight increase in size. The appearance of some aggregations in the nanoparticles may refer to the relatively larger size and hydrogen bonding between the particles^63^. It stimulates the release of the encapsulated compound, so it should be centrifuged immediately after nanoparticle synthesis or up to 30 min. at 5000 rpm. The dynamic light scattering (DLS) evaluates the particle size for the solution of the NPs. This remark can be correlated to delivering an image for a definite area for measurement via TEM, whereas DLS gives an overall observation of the nanoparticles and their agglomeration. In addition, DLS measurement provides a hydrodynamic radius of nanoparticles (hydrated and swollen particles) in aqueous solution. On the other hand, TEM provides the diameter of dried nanoparticles. Zeta potential reveals the positive charge of the synthesized CD NPs due to the cationic nature of the amine groups in the protonated form, resulting from the acidic solvent medium for chitosan^64^. It indicates highly stable nanoparticles of chitosan/dextran sulphate. The XRD analysis showed that pure chitosan has a high degree of crystallinity, defined by distinct peaks at (2θ) of 20 and 10 degrees, corresponding to the crystallographic planes (110) and (020), respectively, which pertain to the non-deacetylated component of chitosan (chitin)^65^. The crystallinity of nano chitosan significantly diminished due to cross-linking with dextran sulfate during the synthesis of chitosan nanoparticles, signifying the amorphous characteristics of these nanoparticles^66^. Chitosan nanoparticles exhibited a prominent diffraction peak at 2θ values of 20.48 Å, characteristic of chitosan nanoparticles^67^. CD-TGC resulted in a lower MIC and caused the downregulation of efflux pump genes *ram*A and *acr*B. Similarly, Wang et al.^68^ employed D-alpha tocopheryl polyethylene glycol succinate-modified and S-thanatin peptide-functionalized nanorods derived from calcium phosphate nanoparticles to deliver TGC for treating pneumonia induced by TGC-resistant *Klebsiella pneumoniae*, resulting in the downregulation of *acr*A, *acr*B, and *ram*A efflux pump genes.

Moreover, Elbi et al..^61^ examined CIP-loaded chitosan nanoparticles (cCNPs) and fucoidan-coated cCNPs (Fu-cCNPs) in their efficacy against *Salmonella* Paratyphi A. The intercellular anti-*Salmonella* efficacy of Fu-cCNPs was twofold greater than cCNPs and sixfold greater than CIP alone. Nevertheless, they did not examine the expression of efflux pump genes. Another study used the antipsychotic medications chlorpromazine and amitriptyline as inhibitors of the AcrB transporter, a crucial component of the prominent RND efflux pumps in *S.* Typhimurium by interfering with substrate binding^69^.

Nonetheless, the anti-efflux properties of chemical agents like carbonyl cyanide 3-chlorophenylhydrazone (CCCP), an efflux pump inhibitor, against CIP- and TGC-resistant *Salmonella* isolates led to a substantial decrease in their MIC values and reinstated their susceptibility to CIP and TGC^8^. Moreover, Razavi et al.^70^ reported a decrease in CIP and TGC MICs following the use of the CCCP EPI. In contrast to these studies, we used natural CD nanocapsules for drug delivery, which are safe and eco-friendly.

The time-kill study indicates that the CD-TGC has a rapid killing activity and exhibits robust and efficient antimicrobial effects, leading to a pronounced reduction in bacterial colony counts at both high and low inoculum levels. The CD-TGC proved efficacious in treating infections caused by *S.* Typhimurium in mice models. This was evident through the high survival rate (100%) compared to the TGC group (40%). Also, *S.* Typhimurium burden in organs decreased as there was a substantial reduction (*P* < 0.05) in the colony count in both unloaded CD NPs and CD-TGC treated groups in both liver (3.6 and 2.6 log10) and intestine samples (3.7 and 2.7 log10) compared to the positive control which was 4.8 log10 in liver and intestine samples.

Concerning liver and kidney functions, *S.* Typhimurium-infected mice showed increases in ALT, ALP, AST, creatinine, urea, LDH, Uric acid, total bilirubin, TP, and Alb. Albumin and TP were markedly reduced in the group receiving TGC than the positive control and improved significantly in both unloaded CD NPs and CD-TGC treated groups (*p* < 0.05). The activities of ALT and LDH were markedly lower in the group treated by CD-TGC compared to the positive control. The levels of sera creatinine, urea, and uric acid levels were markedly reduced in all treated groups relative to the positive control (*p* < 0.05), minimizing in the CD-TGC group. This indicates the stressful effects of *Salmonella* infection on hepatic and renal tissues. The impairment of the functions of these organs was also reported by Seo et al.^71^ who recorded significant elevations of AST and LDH. Additionally, the elevation of liver enzyme ALT and reduction in albumin is corroborated by prior research, which indicated that *Salmonella* spp. infection leads to hepatic granulomas or paratyphoid nodules, resulting in the release of liver enzymes into the serum and diminishing the liver’s capacity to synthesize albumin^72^. Treatment of *S.* Typhimurium-infected mice with unloaded CD NPs or CD-TGC protected the liver and kidney from the stressful effects of *Salmonella*. These results may have been due to the antimicrobial and hepatoprotective effects of chitosan nanoparticles, exhibiting antioxidant and anti-inflammatory properties^73^. Regarding hematological findings, the levels of blood Hb were markedly decreased in the positive control group, while it was significantly higher in CD-TGC, unloaded CD NPs, and TGC groups. The WBC counts, as well as the ratios of neutrophils and lymphocytes, significantly decreased (*p* < 0.05) in all treated groups compared to the positive control. *S.* Typhimurium infection markedly reduced the average amount of hemoglobin due to the declined bone marrow activity and hemophagocytosis^74,75^. Moreover, the significant difference in the elevated neutrophil counts between the untreated and treated groups could be explained by the role of neutrophils in combating salmonellosis^76^. A robust inflammatory stimulus elicits heightened neutrophil production, resulting in left-shift neutrophilia due to the release of neutrophils into circulation^77^. Mice are considered an excellent model for studying the pathogenesis and histological alterations of *Salmonella* spp., and *S*. Typhimurium exhibited evidence of histological damage^78^. As previously reported, the pathological changes caused by *Salmonella* infection are primarily inflammatory in the liver and include congestion, edema, and inflammatory cell infiltrations^79^. The intestinal necrotic alterations are consistent with Lee et al..^80^ The results of this investigation demonstrated a significant reduction in the intensity of the inflammatory response in the groups treated with unloaded CD NPs and CD-TGC. This result is explained by the antibacterial properties of TGC and the anti-inflammatory properties of chitosan NPs^81^, which diminish the intensity of inflammation brought on by salmonellosis. Overall, the *vivo* study demonstrated the safety and effectiveness of CD-TGC in treating MDR *Salmonella* infections, allowing it to be used as a substitute antimicrobial, particularly in cases of bacterial infections that are resistant to antibiotics.

## Conclusions

This study focused on MDR non-typhoidal *Salmonella enterica* species against which TGC demonstrated limited efficacy. This is the first demonstration of anti-efflux and gene-downregulating effects of CD-TGC nanocapsules on MDR *Salmonella enterica*. Our findings demonstrate that CD-TGC nanocapsules serve as an effective drug delivery system for treating intracellular *Salmonella* infections. This approach has the potential to mitigate the development of antimicrobial resistance. CD-TGC demonstrated superior treatment outcomes against Salmonellosis, with a 100% survival rate in treated mice compared to 40% for TGC. This highlights its potential in combating the infection. Further research is warranted to explore the potential of drug-loaded CD nanocapsules for treating infections caused by other pathogens.

## Supplementary Information

Below is the link to the electronic supplementary material.

Supplementary Material 1

## Data Availability

Sequence data supporting this study’s findings have been deposited in the GenBank database’s sequence read archive (SRA) with BioProject accession number PRJNA1189127.
