# Supplementary material for: Chitosan-dextran sulfate nanocapsules for enhanced tigecycline efficacy against non-typhoidal Salmonella enterica
Source: Sci Rep. 2026 Feb 4;16:5016. doi: 10.1038/s41598-026-35229-7 (PMC12876856; doi:10.1038/s41598-026-35229-7)
Supplement: Supplementary file 1 — Supplementary Material 1 [file 41598_2026_35229_MOESM1_ESM.docx]

**
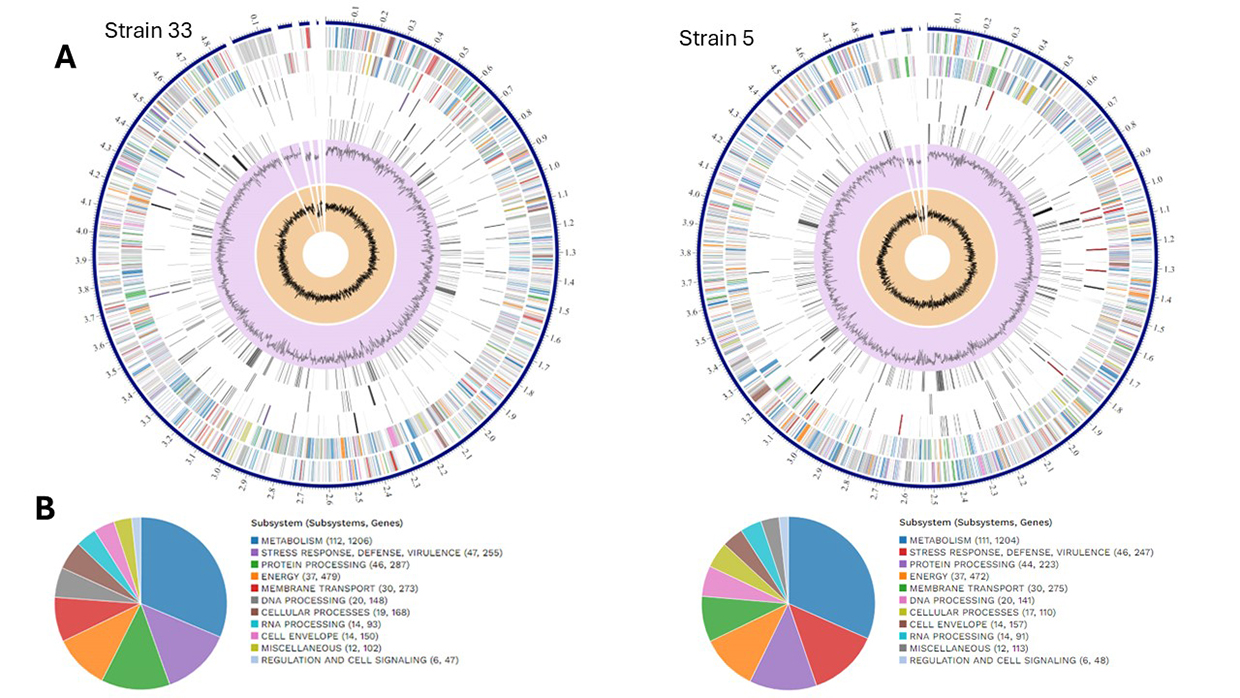
**

**Figure S1**: A circular graphical display of the distribution of the genome annotations of *Salmonella* Bredeney strains 33 (accession no [SRR31606420](https://trace.ncbi.nlm.nih.gov/Traces?run=SRR31606420) ) and 5 (accession no [SRR31606421](https://trace.ncbi.nlm.nih.gov/Traces?run=SRR31606420)). This includes, from outer to inner rings, the contigs, CDS on the forward strand, CDS on the reverse strand, RNA genes, CDS with homology to known antimicrobial resistance genes, CDS with homology to know virulence factors, GC content and GC skew (A). The colors of the CDS on the forward and reverse strand indicate the subsystem that these genes belong to (B).
